# Multi-layered engineering of *Aspergillus terreus* enhances biosynthesis of the plant-derived fungicide physcion

**DOI:** 10.1186/s12934-025-02857-z

**Published:** 2025-11-13

**Authors:** Zilin Ren, Yingying Xue, Ning Xu, Dandan Feng, Ce Geng, Yongsong Wu, Dan Liu, Linshui Zhao, Xiaoxi Zhang, Honglei Ma, Xuenian Huang, Feifei Qi, Xuefeng Lu

**Affiliations:** 1 Shandong Provincial Key Laboratory of Synthetic Biology, Qingdao Institute of Bioenergy and Bioprocess Technology, Chinese Academy of Sciences, No. 189 Songling Road, Shandong 266101 Qingdao, China; 2 Key Laboratory of Biofuels, Qingdao Institute of Bioenergy and Bioprocess Technology, Chinese Academy of Sciences, Qingdao, 266101 Shandong China; 3 Shandong Energy Institute, Qingdao, 266101 Shandong China; 4 Qingdao New Energy Shandong Laboratory, Qingdao, 266101 Shandong China; 5 School of Biological Science and Technology, University of Jinan, Jinan, 250022 Shandong China; 6 College of Life Science, Dalian Minzu University, Dalian, 116600 Liaoning China; 7 University of Chinese Academy of Sciences, Beijing, 100049 China; 8 Marine Biology and Biotechnology Laboratory, Qingdao National Laboratory for Marine Science and Technology, Qingdao, 266237 Shandong China

**Keywords:** Physcion, Emodin, Aspergillus terreus, Microbial cell factory, Cytochrome P450 enzyme, Synthetic biology, Detoxification

## Abstract

**Background:**

Emodin and its derivatives are important bioactive anthraquinones from rhubarb, with diverse pharmacological activities. Physcion, an *O*-methylated derivative of emodin, is a promising plant-derived fungicide and pharmaceutical lead. However, plant extraction yields are low and land-intensive, while microbial production is hampered by inefficient conversion and byproduct accumulation.

**Results:**

Here, we identify a cytochrome P450 enzyme (CYP-H6231) that, with its dedicated redox partner cytochrome P450 reductase (CPR-H10273), converts emodin to ω-hydroxyemodin in *Aspergillus terreus*. Deletion of CYP-H6231 increased physcion titer by 1.8-fold and significantly improved product purity. Further engineering, via 3-*O*-methyltransferase overexpression, SAM pathway enhancement, and enzyme fusion, yielded only modest improvement (up to 37%), likely due to compromised strain robustness from the loss of CYP-H6231 mediated detoxification. Structural modeling and mutagenesis of CYP-H6231 revealed key residues for substrate recognition and catalysis.

**Conclusions:**

This study reveals a detoxification bottleneck in anthraquinone biosynthesis and establishes two improved *A. terreus* platforms for scalable production of physcion and emodin, respectively, highlighting trade-offs between pathway efficiency and cellular fitness.

**Supplementary Information:**

The online version contains supplementary material available at 10.1186/s12934-025-02857-z.

## Background

In recent decades, the field of synthetic biology has witnessed significant advancements, particularly in the area of microbial production of plant natural products and commodity chemicals. This emerging field holds great promise in various industries including fuels, chemicals, medicines, and environmental protection [1–4].

Emodin (compound 1) and its derivatives play a crucial role as bioactive compounds in traditional Chinese medicine rhubarb. These anthraquinone compounds exhibit diverse bioactivities, making them valuable in clinical research for their potential in antitumor, antiviral, antioxidant, anti-inflammatory, and constipation treatment applications [5, 6]. Notably, one of the derivatives called physcion (compound 2), which is an *O*-methylated derivative of emodin at the C3 hydroxyl group, has been successfully developed into a fungicide and is currently available in the Chinese market [7–9].

Like other plant-derived natural products, rhubarb-based production of emodin and its derivatives requires substantial land, water, and time. Its reliance on agricultural biomass introduces supply variability due to factors such as pests, weather extremes, and other environmental stresses, while complex cultivation and extraction processes constrain scalability and energy efficiency, hindering green manufacturing.

Emodin is a significant intermediate in the biosynthesis of various fungal seco-anthraquinone [10, 11]. Establishing microbial factories using synthetic biology approaches to sustainably and efficiently produce emodin and physcion on a large scale is a promising strategy [12, 13]. For instance, a *Saccharomyces cerevisiae* cell factory achieved an emodin titer of 528.4 ± 62.7 mg/L after 5 days of fed-batch fermentation [14]. The Liu’s group also identified some *O*-methyltransferases and developed a physcion-producing mutant strain of *Aspergillus ninulans*, achieving a maximum yield of 64.6 mg/L in shake-flask fermentation [15].

In our previous study, a first-generation physcion-producing cell factory was constructed by introducing an emodin-3-OH-*O*-methyltransferase (3-EOMT) gene into an emodin-accumulating mutant. This resulted in a maximum titer of 6.3 g/L after 14 days of fed-batch fermentation in a 100-liter bioreactor [9, 16].

**Fig. 1:**
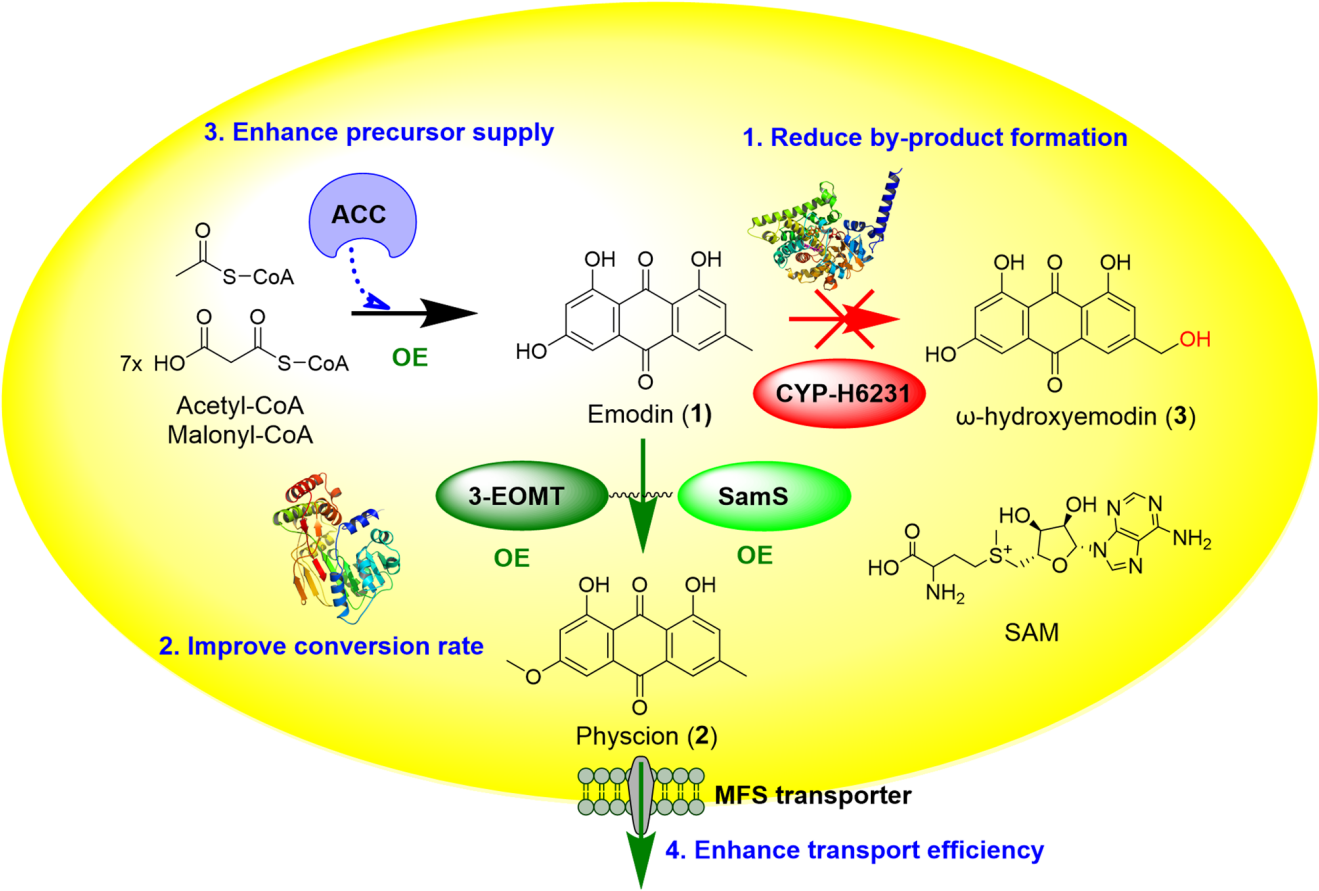
Engineering strategies for enhanced physcion production in second-generation *A. terreus* cell factory. Multiple approaches were employed including: (1) minimizing hydroxylated by-product formation through CYP-H6231 knockout, (2) improving conversion efficiency via 3-emodin-*O*-methyltransferase (3-EOMT) and S-adenosylmethionine synthase (SamS) overexpression, (3) enhancing precursor supply through acetyl-CoA carboxylase (ACC) overexpression, and (4) enhancing membrane transport via MFS transporter overexpression. OE: overexpression. Compound 1, emodin; compound 2, physcion; and compound 3, ω-hydroxyemodin

However, the accumulation of intermediate emodin and byproducts such as ω-hydroxyemodin (compound 3) and fallacinol (compound 4) significantly decreased physcion yield and purity, increasing downstream processing burdens and production costs, and thus hindering commercial development. To overcome these bottlenecks, we applied multiple metabolic engineering strategies to construct a series of improved *A. terreus* cell factories with enhanced physcion productivity and purity (Fig. 1). Furthermore, the critical cytochrome P450 enzyme (CYP) H6231, responsible for detoxification in *A. terreus*, along with its dedicated cytochrome P450 reductase (CPR) H10273 (Table S1), was identified. Using AlphaFold2, substrate docking, and mutation assays, the emodin binding sites and key catalytic residues of CYP-H6231 were elucidated. This detailed understanding of the catalytic mechanism of this essential CYP enzyme in *A. terreus* provides valuable insights into anthraquinone detoxification, thereby enabling the construction of more efficient cell factories for green and scalable physcion production and offering practical strategies for balancing pathway optimization with host fitness.

## Materials and methods

### Strains, chemicals and general DNA manipulation procedures

The parent strain consisted of *A. terreus* HXN301, a statin-producing strain; ΔgedA, an emodin-accumulating variant; and PgedA-PtaI, a physcion-producing variant, all of which were stored in our laboratory (Table S2). Authentic standards of emodin and physcion were purchased from Solarbio (Beijing, China). ω-hydroxyemodin was prepared from the fermentation culture of ΔgedA and confirmed by NMR analysis. FastDigest restriction enzymes (Thermo Fisher Scientific, Vilnius, Lithuania) and the ClonExpress Ultra One-Step Cloning Kit (Vazyme, China) were used for vector construction following the manufacturer’s instructions. Fungal genomic DNA and plasmids were extracted using the Fungal DNA Kit and Plasmid Mini Kit I (Omega Bio-Tek, Norcross, USA), respectively. The Gel Extraction Kit and Cycle Pure Kit, used for agarose gel and PCR product purification, were also procured from Omega Bio-Tek. Oligonucleotide synthesis and DNA sequencing services were carried out by TsingKe Biotech and WeiLai Biotech (Qingdao, China), while DNA synthesis was performed by BGI Genomics (Beijing, China). The oligonucleotide primers used are listed in Table S3. *Escherichia coli* DH5α and BL21 (DE3) were used separately for gene cloning and protein expression at 37 °C in Lysogeny Broth (LB) medium supplemented with appropriate antibiotics. *S. cerevisiae* BY4742 and plasmid pESC-HIS were kindly gifted by Professor Yijian Rao [17].

### Construction of the ΔgedA-ΔpyrG and PgedA-PtaI-ΔpyrG strains

*A. terreus* ΔgedA and PgedA-PtaI were constructed according to the methodology previously exemplified [9, 16]. The upstream and downstream sequences of *pyrG*_*An*_ were amplified from the genomic DNA of *A. terreus* HXN301 using the primer pairs UpyrGAn-F/UpyrGAn-R and DpyrGAn-F/DpyrGAn-R, respectively, and subsequently joined by fusion PCR to generate the final deletion cassette. In addition, the gene-targeting construct was amplified using primers CpyrGAn-F/CpyrGAn-R and transformed into ΔgedA and PgedA-PtaI. Transformants were selected on CDS plates (3 g/L NaNO_3_, 2 g/L KCl, 1 g/L KH_2_PO_4_, 0.5 g/L MgSO_4_·7H_2_O, 0.5 g/L FeSO_4_·7H_2_O, 10 g/L glucose, 1.2 M sorbitol and 1.5% agar) containing 5 mM uracil, 5 mM uridine and 5 mM 5-fluoroorotic acid (5-FOA). Selected transformants were further purified through single-spore isolation, and their genotypes were verified by genomic PCR using the primer pair Uan-F/Dan-R and gene sequencing.

### Construction of CPR-H10273, CYP-H6231, CYP-H2247 and CYP-H3979 deletion variants in ΔgedA-ΔpyrG and PgedA-PtaI-ΔpyrG

To delete hypothetical hydroxylase-related genes *CPR-H10273*, *CYP-H6231*, *CYP-H2247* and *CYP-H3979* in the ΔgedA-ΔpyrG strain, approximately 1.0 kb of both the 5’ and 3’ flanking regions of each target gene were amplified from the *A. terreus* HXN301 genomic DNA using the following primer pairs: U10273-F/U10273-R and D10273-F/D10273-R for *CPR-H10273*, U6231-F/U6231-R and D6231-F/D6231-R for *CYP-H6231*, U2247-F/U2247-R and D2247-F/D2247-R for *CYP-H2247*, U3979-F/U3979-R and D3979-F/ D3979-R for *CYP-H3979*. The *pyrG*_*An*_ fragment was amplified using pyrGAn-F/pyrGAn-R. The upstream, *pyrG*_*An*_, and downstream fragments were fused via fusion PCR and further amplified using the following primer pairs: C10273-F/C10273-R, C6231-F/C6231-R, C2247-F/C2247-R, and C3979-F/C3979-R. The resulting PCR products were transformed into the ΔgedA-ΔpyrG strain. Transformants were regenerated on Potato Dextrose Agar (PDA) plates (4 g/L potato starch, 25 g/L dextrose, and 15 g/L agar) supplemented with 1.2 M sorbitol (Solarbio, China), and their genotypes were confirmed using genomic PCR with primer pairs U10273-F/D10273-R, U2247-F/D2247-R, U3979-F/D3979-R, U6231-F/pyrG-in-R, and pyrG-in-F/D6231-R. *CYP-H6231* was deleted in the PgedA-PtaI-ΔpyrG strain and validated using the same strategy as described for ΔgedA-ΔpyrG.

### Construction of 3-EOMT, SamS, ACC and EwMFS overexpression variants

Gene expression was driven by either the native promoter of *CYP-H6231* or the constitutive strong promoter P*gpdAt*. Specifically, the flanking sequences of *CYP-H6231* were amplified from the *A. terreus* HXN301 genome using primer pairs U6231-F/U6231-R and D6231-F/D6231-R. The P*gpdAt* promoter was amplified from the pXH102 plasmid with primer pair PgpdAt-6231-F/PgpdAt-R [18]. The T*tef* terminator was amplified from the pFC332 plasmid [19] using primer pair Ttef-F/Ttef-R. The *pyrG*_*An*_ fragment was amplified using primer pair pyrGAn-loxP-F/pyrGAn-loxP-R, while the *PtaI* fragment was amplified from pET-28b-PtaI plasmid. Additionally, the *AwOMT* and *EwMFS* fragments were synthesized by BGI Genomics (Beijing, China). The *SamS* gene fragment was amplified from the cDNA of HXN301 following the previously reported protocol [9, 16]. An *ACC* gene fragment was amplified using the genomic DNA of *A. terreus* HXN301 as the template. Each of these genes (*PtaI*, *AwOMT*,* EwMFS*,* SamS* and *ACC*) was individually assembled with the 5’ and 3’ flanking sequences of *CYP-H6231*, the *pyrG*_*An*_ fragment, and the T*tef* terminator. Depending on the construct, the P*gpdAt* promoter was either included or omitted before gene insertion. All these fragments were integrated into the pUC19 vector via one-step cloning. Several integration constructs were constructed, comprising the following sequences: U6231-PgpdAt-PtaI-Ttef-pyrG_An_-D6231, U6231-PtaI-Ttef-pyrG_An_-D6231, U6231-PgpdAt-AwOMT-Ttef-pyrG_An_-D6231, U6231-AwOMT-Ttef-pyrG_An_-D6231, U6231-PgpdAt-SamS-Ttef-pyrG_An_-D6231, U6231-SamS-Ttef-pyrG_An_-D6231, U6231-PgpdAt-EwMFS-Ttef-pyrG_An_-D6231 and U6231-PgpdAt-ACC-Ttef-pyrG_An_-D6231. The assembled constructs were amplified using the primers C6231-F/C6231-R and subsequently transformed into the uracil auxotrophic *A. terreus* variant PgedA-PtaI-ΔpyrG. Homologous recombination was employed to restore the *pyrG*_*An*_ gene, resulting in transformants where the *CYP-H6231* locus was replaced with the overexpression constructs.

The transformants were regenerated on PDA plates supplemented with 1.2 M sorbitol (Solarbio, China), and genomic PCR using the primer pairs U6231-F/pyrGAn-in-R, pyrGAn-in-F/D6231-R and H6231-in-F/H6231-in-R were performed to verify the correct transformants.

### Construction of variants using protein fusion and co-expression strategy

AwOMT-GedH fusion variants were constructed using both rigid (EAAAK) and flexible (GGGGS) linkers. The two gene fragments were arranged in two different orders: AwOMT-GedH and GedH-AwOMT, leading to four distinct constructs. The fragments U6231-PgpdAt-AwOMT, U6231-PgpdAt, AwOMT-Ttef-pyrG_An_-D6231 and Ttef-pyrG_An_-D6231 were amplified from the plasmid pUC19-6231-PgpdAt-AwOMT-Ttef-pyrG_An_ using primer pairs U6231-F/AwOMT-R, U6231-F/PgpdAt-R, AwOMT-F/D6231-R and Ttef-F/D6231-R, respectively. To integrate GedH, four distinct primer pairs, AwOMT-EAAAK-GedH-F/Ttef-GedH-R, PgpdA-GedH-F/AwOMT-EAAAK-GedH-R, AwOMT-GGGGS-GedH-F/Ttef-GedH-R and PgpdA-GedH-F/AwOMT-GGGGS-GedH-R, were used to amplify the gene from *A. terreus* HXN301. Each fragment was ligated into the pUC19 vector via one-step cloning, generating four overexpression cassettes: U6231-PgpdAt-AwOMT-EAAAK-GedH-Ttef-pyrG_An_-D6231, U6231-PgpdAt-AwOMT-GGGGS-GedH-Ttef-pyrG_An_-D6231, U6231-PgpdAt-GedH-EAAAK-AwOMT-Ttef-pyrG_An_-D6231 and U6231-PgpdAt-GedH-GGGGS-AwOMT-Ttef-pyrG_An_-D6231. For the construction of the IGG6-SamS fusion variant, a fragment containing *SamS* fused with *IGG6* was amplified from pUC19-U6231-PgpdAt-SamS-Ttef-pyrGAn-D6231 using the primer pair AwOMT-IGG6-SamS-F/Ttef-SamS-R. This fragment was integrated into pUC19 along with U6231-PgpdAt-AwOMT and Ttef-pyrGAn-D6231, generating the final cassette U6231-PgpdAt-AwOMT-IGG6-SamS-Ttef-pyrGAn-D6231. All recombinant plasmids were amplified with C6231-F/C6231-R primers and transformed into the uracil auxotrophic *A. terreus* variant PgedA-PtaI-ΔpyrG. The transformants were selected and verified as described previously.

### Shake-flask cultivation and titer analysis of Emodin and physcion in secondary metabolites (SMs) from variants

The variants were cultivated on PDA solid plates at 28 ℃ for 7–8 days. Conidia were harvested from a single plate using sterile distilled water. Then, a suspension of 1.0 × 10^7^ to 2.0 × 10^7^ spores were inoculated into 250 mL of Liquid Production Medium (LPM) fermentation medium (70 g/L glucose, 40 g/L sucrose, 1 g/L yeast extracts, 1 g/L peptone, 1 g/L sodium acetate, 2 g/L sodium citrate, 0.04 g/L KH_2_PO_4_, 0.5 g/L PEG-400, 0.1 g/L MgSO_4_·7H_2_O, 1.5 g/L CaCO_3_, pH 6.6 ± 0.3). The conidia were incubated in the medium at 28 °C with rotary shaking at 220 rpm for 7 days. Following incubation, a 1 mL aliquot of the microbial culture broth was collected and centrifuged. The supernatant was discarded, and the mycelial pellets were retained for subsequent weighing. Extraction was performed sequentially with two 10 mL aliquots of CH_2_Cl_2_/MeOH (1:1). After the organic solvent was evaporated, the extract was redissolved in MeOH to a final concentration of 100 mg/mL. Subsequently, the solution was filtered through a 0.22 μm organic membrane, and a 10 µL aliquot was subjected to high-performance liquid chromatography (HPLC) analysis. The HPLC solvent gradient was set as follows: 0–1 min, 50% A/50% B; 1–20 min, 50% A/50% B to 0% A/100% B; 20–24 min, 0% A/100% B; 24–25 min, 0% A/100% B to 50% A/50% B; 25–30 min, 50% A/50% B. Solvent A consisted of 100% H₂O with 0.1% trifluoroacetic acid (TFA, Sigma-Aldrich, St. Louis, MO, USA), while solvent B was 100% ACN with 0.1% TFA.

### Extraction and isolation of SMs from the PgedA-PtaI variant

For the isolation and purification of SMs, HPLC experiments were conducted on a Hitachi Primaide 1110 system equipped with a 1430 diode array detector. For analytical HPLC, a C18 column (Hypersil BDS C18, 4.6 × 250 mm, 5 μm) was used at a flow rate of 1 mL/min, while preparative HPLC collection was conducted using a C18 column (Waters, Symmetry C18, 7.8 × 100 mm, 5 μm) with a flow rate of 3 mL/min. Organic extracts from the PgedA-PtaI variant were purified by semipreparative HPLC with a solvent gradient system (0–5 min, 60% A / 40% B; 5–11 min, 40% A / 60% B to 0% A / 100% B; 11–13 min, 0% A / 100% B; 13–13.1 min, 0% A / 100% B to 60% A / 40% B; 13.1–15 min, 60% A / 40% B), where solvent A consisted of 100% H₂O with 0.1% TFA, and solvent B consisted of 100% ACN with 0.1% TFA, to isolate ω-hydroxyemodin (4.6 min), fallacinol (7 min), emodin (8.6 min), and physcion (12 min).

### Gene synthesis, expression, purification and in vitro enzymatic assays of AwOMT

The *AwOMT* gene was optimized and synthesized according to the codon usage biases of both *E. coli* and *A. terreus* and then inserted into the pET28b vector via BGI Genomics’ gene synthesis service. The constructed plasmid was confirmed by DNA sequencing and transformed into *E. coli* BL21(DE3) for the expression of His_6_-tagged recombinant proteins. The seed culture was transferred (1:100) into 1 L of LB broth containing 5% glycerol, 50 mg/L kanamycin and a rare salt solution and grown at 37 °C until the OD_600_ reached 0.6. Subsequently, 0.2 mM isopropyl β-D-thiogalactopyranoside (IPTG, Solarbio, Beijing, China) was added to the culture to induce protein expression. The culture was maintained at 16 °C with rotary shaking at 220 rpm for an additional 20 h. AwOMT protein was purified using Ni-NTA affinity chromatography as previously described [20]. The concentration of the AwOMT protein was determined using Bradford Protein Assay Kit (Beyotime Biotechnology, Shanghai, China) based on a standard curve of bovine serum albumin. The standard AwOMT assay contained 2 µM protein, 200 µM emodin, and 1 mM S-adenosylmethionine (SAM) in a 100 µL reaction buffer (50 mM NaH_2_PO_4_, pH 7.4). The reaction was performed for 8 h at 30 °C and quenched by extraction with 400 µL ethyl acetate. The upper organic layer was collected, dried under a gentle nitrogen stream, and redissolved in 200 µL of methanol. Following centrifugation at 15,871 × g for 15 min, a 20 µL aliquot of the sample was analyzed by HPLC.

### Transcriptome analysis for anthraquinone hydroxylase identification and gene knockout characterization

A total of 1.67 × 10^7^ spores of the PgedA-PtaI strain were inoculated into 50 mL of LPM fermentation medium and cultivated for 1, 2, 4, and 6 days, with three biological replicates per time point. Mycelia were collected via centrifugation at each time point for RNA sequencing and titer determination. The physcion titer was quantified using a standard calibration curve and presented as a time-course curve. A control experiment was conducted with a 1-day culture of the parental strain HXN301, which harbors an inactive geodin biosynthetic gene cluster (BGC) and was processed identically for comparative RNA sequencing. Transcriptome sequencing was performed using the Illumina platform to produce raw reads. The quality of raw reads was assessed using FastQC. Clean reads were obtained by removing low-quality reads and adapter sequences, and were subsequently used for downstream analyses. Clean reads were mapped to the reference genome using HISAT2. Gene expression levels were quantified in terms of FPKM (Fragments Per Kilobase of transcript per Million mapped reads) using StringTie. Differential expression analysis was performed with DESeq2. FPKM values of all CYP genes from the PgedA-PtaI samples were extracted and compared with those from the HXN301 control sample (1-day sample) to identify differentially expressed CYP genes (Table S4).

### Emodin and SAM feeding assays

To perform an emodin feeding assay, approximately 1 × 10⁷ spores of *A. terreus* HXN301 were inoculated into 30 mL of LPM medium and cultured at 28 °C with shaking at 220 rpm. After 3 days of fermentation, emodin was added to the culture broth at a final concentration of 100 µM. Fermentation was continued for an additional 1–2 days before sample collection. Culture extracts were analyzed by HPLC to detect the presence of ω-hydroxyemodin.

To evaluate the effect of SAM supplementation on product biosynthesis, SAM was added to the HXN301 culture at final concentrations of 0 (negative control), 1, 5, or 10 mM on day 3 post-inoculation. Samples were collected on days 5, 6, and 7 for quantitative analysis of emodin and physcion production. Titers were determined by HPLC, and each experiment was performed in triplicate.

### Cryo-scanning electron microscopy analysis of spores

The spore suspension was centrifuged to collect the cells, followed by repeated rinsing with PBS (pH 7.2–7.4) to remove residual contaminants. The samples were fixed in 2.5% glutaraldehyde for 1 h, followed by additional washes with PBS. After stepwise dehydration with ethanol and tert-butanol, the samples were immersed in tert-butanol and lyophilized until fully dehydrated. Finally, the samples were mounted on a sample holder, coated with a thin layer of metal by ion sputtering, and imaged using a field-emission scanning electron microscope (Hitachi S-4800, Hitachi High-Technologies, Tokyo, Japan).

### High-resolution mass spectrometry

LC-HRMS analysis was performed using a Waters Symmetry C18 column (5 μm, 2.1 × 150 mm) with electrospray ionization (ESI) in both positive and negative modes. The mobile phase gradient was as follows: 0–1 min, 40% A/60% B; 1–18 min, 40% A/60% B to 0% A/100% B; 18–24 min, 0% A/100% B; 24–25 min, 0% A/100% B to 40% A/60% B; 25–30 min, 40% A/60% B, at a flow rate of 0.2 mL/min (solvent A: H₂O with 0.1% formic acid; solvent B: ACN). HRESIMS data were acquired on a Dionex Ultimate 3000 system coupled to a Bruker Maxis Q-TOF. The ion source parameters were as follows: dry temperature, 180 °C; drying gas flow, 6 L/min; nebulizer pressure, 1.0 bar; and capillary voltage, 4.5 kV (positive mode) and 2.2 kV (negative mode).

### Molecular docking

The structural model of CYP-H6231 was predicted by AlphaFold2 [21]. The CYP-H6231 and heme complex was constructed by structural alignment with P450BM3 (PDB: 3WSP), and the heme coordinates were transferred accordingly. Hydrogen atoms were added to both the protein and the heme. A binding site sphere with a 10 Å radius centered on the heme iron atom was defined for docking. Emodin was docked into the CYP-H6231 and heme complex using Discovery Studio, and the pose with the lowest binding energy was selected for analysis.

### Mutagenesis of *CYP-H6231*, expression in *S. cerevisiae*, and enzymatic activity analysis

The intron-free *CPR-H10273* fragment was subdivided into two segments and amplified from the HXN301 genome using the primer pairs 10,273-e1-F/10,273-e1-R and 10,273-e2-F/10,273-e2-R. Simultaneously, the *CYP-H6231* fragment was amplified using the primer pair 6231-F/6231-R. The pESC-derived expression vector, containing both *CYP-H6231* and *CPR-H10273*, was successfully constructed via one-step cloning and verified by genomic PCR using the primer pair pESC-HIS-F/pESC-HIS-R. Site-directed mutagenesis of *CYP-H6231* was performed using PCR-based methods with plasmid pESC-H6231-H10273 as the template, following the standard QuikChange^®^ protocol. All plasmids were confirmed by DNA sequencing. Recombinant plasmids were transformed into *S. cerevisiae* strain BY4742, and transformants were selected on SD agar plates lacking histidine and supplemented with 2% glucose. Verified transformants were pre-cultured in 40 mL SD medium at 28–30 °C with shaking at 220 rpm for 48 h. The cultures were then transferred to 20 mL SG medium supplemented with 50 mM emodin and incubated under the same conditions for another 48 h. A 1 mL aliquot of the culture broth was extracted twice with an equal volume of ethyl acetate using ultrasonic treatment for 30 min. The combined organic layers were then concentrated to dryness. The dried extracts were dissolved in 600 µL of HPLC-grade methanol and filtered through a 0.22 μm organic membrane. Finally, 10 µL of each sample was injected into the HPLC system for component analysis.

## Results and discussion

### Accumulation of hydroxylated derivatives of Emodin and physcion in the first-generation microbial factory

**Fig. 2:**
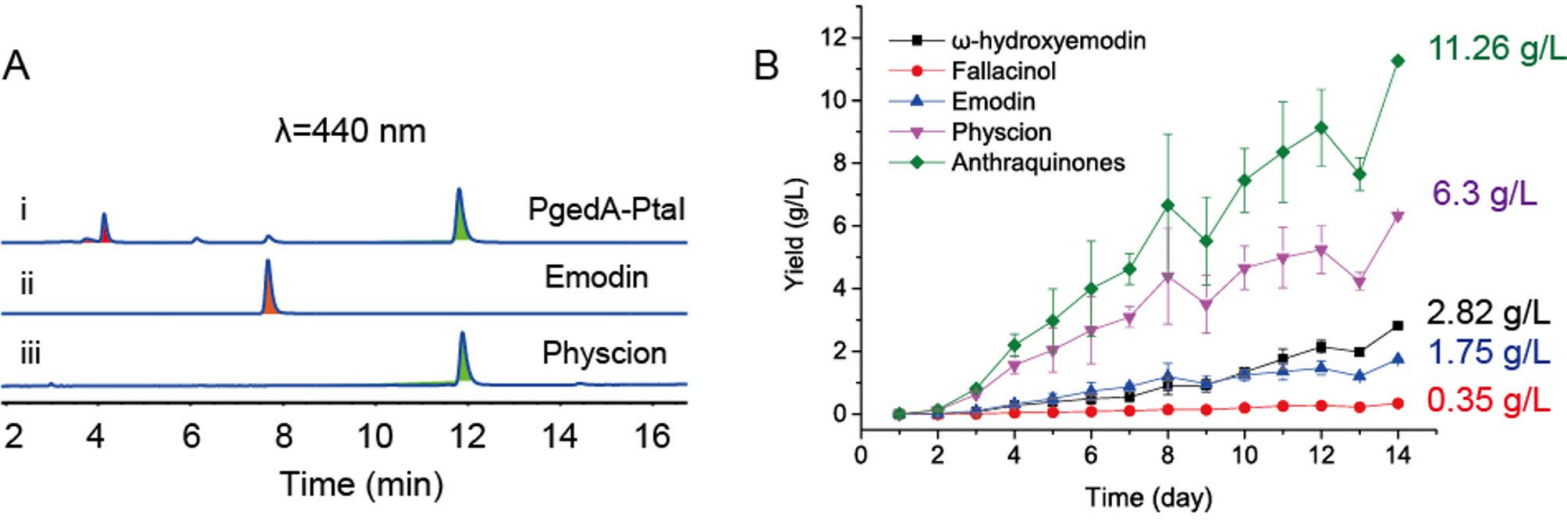
By-products (ω-hydroxyemodin and fallacinol) and intermediate (emodin) yields analysis of physcion producing first-generation variant PgedA-PtaI. (**A**) HPLC analysis of fermented products of the *A. terreus* variant PgedA-PtaI on the fourth day at 440 nm. Traces ii and iii represent the authentic standards of emodin and physcion, respectively. The peaks of ω-hydroxyemodin, emodin and physcion are filled in red, orange and green, respectively. (**B**) The titer time courses of emodin and physcion derivatives during fed-batch fermentation of the PgedA-PtaI in a 100-liter bioreactor. Error bars represent the standard deviations of three identical replicates

In our previous study, the physcion-producing *A. terreus* variant PgedA-PtaI achieved a titer of 6.3 g/L after 14 days of fed-batch fermentation in a 100-L fermentor [9]. However, HPLC analysis of variant PgedA-PtaI revealed two other anthraquinone compounds besides emodin and physcion at 440 nm (Fig. 2A). The *m/z* values of these compounds were 285.0406 ([M–H]⁻, corresponding to [C₁₅H₉O₆]⁻) and 301.0703 ([M + H]⁺, corresponding to [C₁₆H₁₃O₆]⁺), respectively (Fig. S1). To determine their structure, both compounds were prepared on a large scale using semi-preparative HPLC. Combined LC-HRMS and NMR analyses confirmed that the compounds are hydroxylated derivatives of emodin and physcion, specifically ω-hydroxyemodin and fallacinol (Fig. S1). Subsequent titer analysis indicated the presence of approximately 2.82 g/L of ω-hydroxyemodin, 0.35 g/L of fallacinol, and 1.75 g/L for the unconverted precursor emodin (Fig. 2B). These by-products and intermediates together accounted for approximately 43.7% of the total anthraquinone content. To further improve the yield and purity of physcion, it is imperative to eliminate these by-products and enhance the conversion efficiency of emodin.

### Unveiling the types of anthraquinone hydroxylases

To identify and delete the cryptic hydroxylase responsible for the formation of hydroxylated by-products, we examined potential candidates including FAD monooxygenases (FMOs) and CYPs. However, the absence of these enzymes in the geodin BGC suggests that an external gene is involved in this transformation. This aligns with previous findings by Oakley et al., who proposed the involvement of an unknown CYP enzyme in hydroxylating chrysophanol to produce aloe-emodin in the prenyl xanthone biosynthesis pathway of *Aspergillus nidulans* [22]. Thus, a genome scan was conducted based on the study by Kelly and colleagues [23] to identify the complete sets of CYPs and FMOs in *A. terreus* HXN301. The analysis unveiled the presence of 131 CYPs and 38 FMO family proteins.

**Fig. 3:**
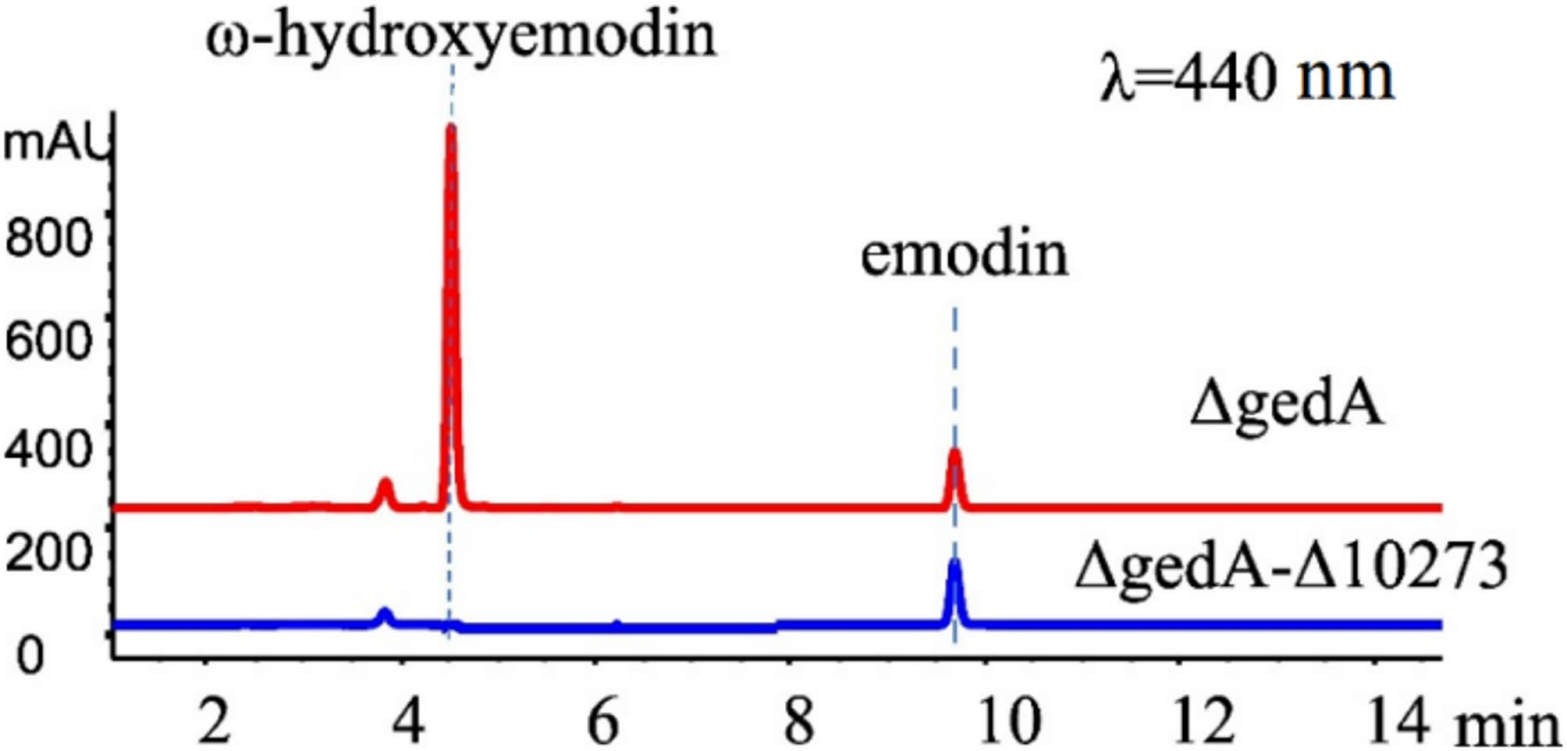
Deciphering the type of anthraquinone hydroxylase. HPLC analysis of fermentation culture extracts of ΔgedA and ΔgedA-Δ10273 on the seventh day at 440 nm

Given the relatively limited number of CPRs compared to the abundance of CYPs and FMOs, we focused on CPRs, as most eukaryotes possess no more than two that can donate electrons to multiple CYPs [24]. Among the seven CPRs identified, only H10273 and H764 were classified as Class II microsomal type A CPRs (Table S5). Based on this, *CPR-H10273* was knocked out in the uridine-deficient emodin-producing strain ΔgedA-ΔpyrG, resulting in the variant ΔgedA-Δ10273. Gratifyingly, deletion of *CPR-H10273* abolished the hydroxylation of emodin. However, emodin titers did not significantly increase, and total anthraquinone accumulation in ΔgedA-Δ10273 notably decreased to about 10% of the parent strain ΔgedA (Fig. 3). Moreover, the deletion of CPR impaired strain growth, likely due to disrupted electron transfer to essential CYPs involved in core metabolic processes. Thus, these results demonstrated that the elusive anthraquinone hydroxylase is a CYP enzyme with CPR-H10273 as its specific reductase, providing valuable insights for future research endeavors.

### Suppression of hydroxylated by-products and identification of the key anthraquinone hydroxylase

To identify the cryptic CYP enzyme among the 131 candidates, initial experiments were conducted to determine whether it is also expressed in the parent strain HXN301, which exhibits extremely low anthraquinone productivity. Unexpectedly, when emodin was fed into the HXN301 culture, a considerable portion of emodin was converted into its hydroxylated derivative (Fig. S2). This finding suggests that the unidentified CYP is expressed robustly, irrespective of anthraquinone production.

**Fig. 4:**
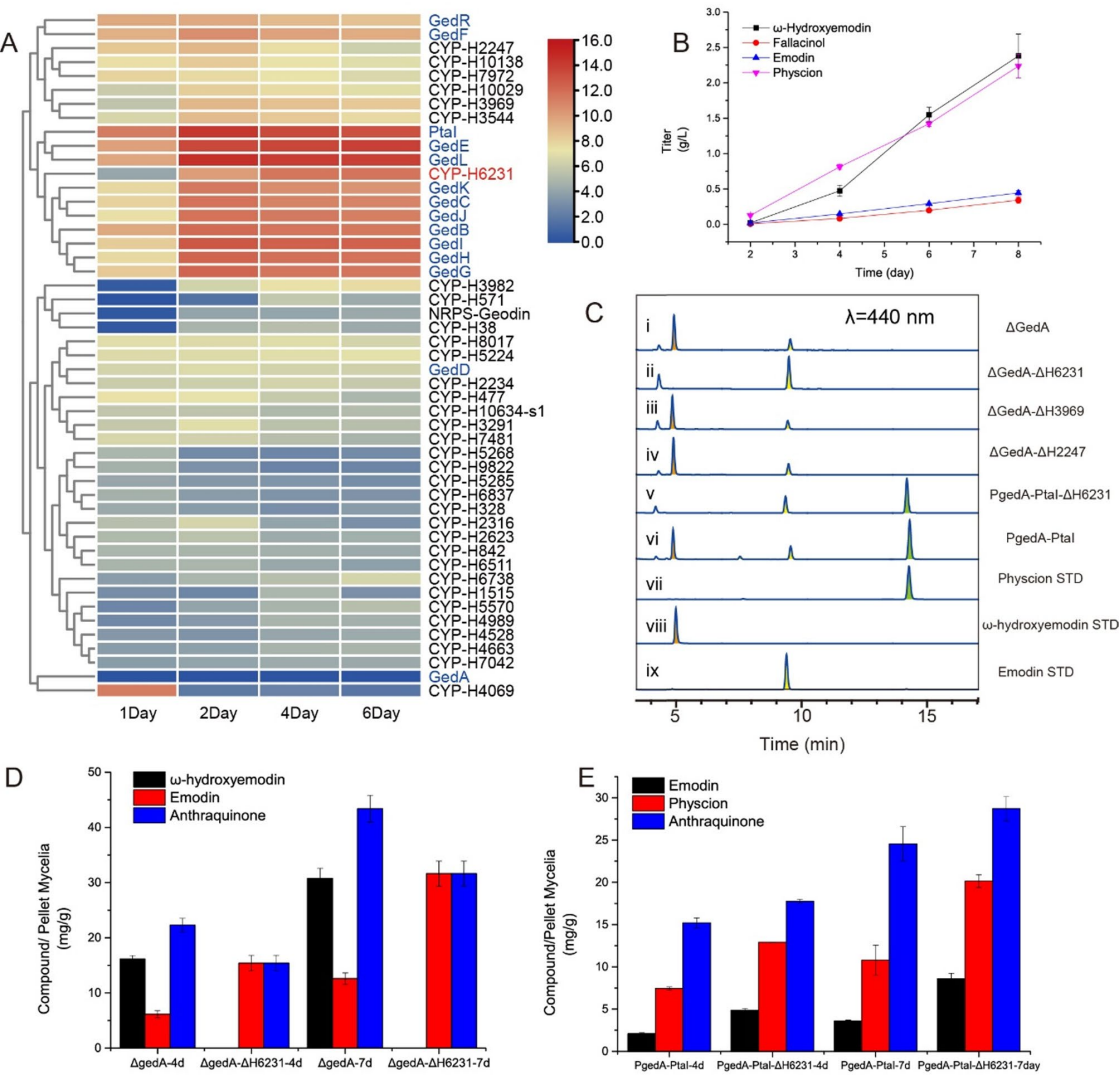
Transcriptome-based identification of anthraquinone hydroxylase, functional gene characterization, and metabolite titer analysis. (**A**) Heatmap illustrating transcript levels of the geodin BGC and 34 CYP genes. (**B**) Time-course accumulation of emodin, ω-hydroxyemodin, physcion, and fallacinol in the PgedA-PtaI strain during transcriptome profiling. (**C**) HPLC chromatograms (440 nm) showing comparative metabolite profiles of gene deletion mutants in the ΔgedA background (i–iv) and the PgedA-PtaI strain (v–vi) on day 4. Standard compounds (vii–ix): physcion (green), ω-hydroxyemodin (orange), and emodin (yellow). (**D**) Comparison of emodin, ω-hydroxyemodin, and total anthraquinone production (mg/g) between ΔgedA and ΔgedA-ΔH6231 strains after 4 and 7 days of cultivation. (**E**) Comparison of emodin, physcion, and total anthraquinone production (mg/g) between PgedA-PtaI and PgedA-PtaI-ΔH6231 strains after 4 and 7 days of cultivation

Transcriptome sequencing of the PgedA-PtaI strain at different time points revealed that 9 CYPs with FPKM > 10 were identified on the second day (Table S4). Heatmap analysis of geodin biosynthetic genes and 34 CYPs suggested that CYP-H6231 could be a promising candidate (Fig. 4A), because it not only clustered with other geodin biosynthetic genes, but its transcription level also significantly increased on the fourth and sixth days, corresponding with a notable rise in ω-hydroxyemodin production during this period (Fig. 4B).

Further investigations involved knocking out the three CYPs with the highest FPKM values (CYP-H6231, CYP-H2247, and CYP-H3969) in the uridine auxotroph of the first-generation emodin-producing strain ΔgedA-ΔpyrG to create three mutants (Fig. S3). Fermentation followed by HPLC analysis confirmed that the deletion of *CYP-H6231* resulted in the complete cessation of ω-hydroxyemodin production, establishing CYP-H6231 as the long-sought anthraquinone hydroxylase (Fig. 4C trace ii). In addition, the emodin titer in the ΔgedA-ΔH6231 strain increased by approximately 2.5-fold on day seven (31.6 mg/g vs. 12.6 mg/g mycelia) compared to the parent strain ΔgedA (Fig. 4D), corresponding to a volumetric titer of approximately 3.8 g/L versus 1.5 g/L (Fig. S4A). Thus, a specific emodin-producing *A. terreus* cell factory, ΔgedA-ΔH6231, has been constructed.

Subsequently, the emodin hydroxylase CYP-H6231 was knocked out in the uridine auxotroph of the first-generation physcion-accumulating cell factory PgedA-PtaI-ΔpyrG to generate the mutant PgedA-PtaI-ΔH6231 (Fig. S3). Further HPLC analysis of the fermentation extract revealed that the production of the hydroxylated derivatives ω-hydroxyemodin and fallacinol was abolished (Fig. 4C trace v). Moreover, as expected, the titer of physcion increased significantly by approximately 1.8-fold on day seven (20.1 mg/g vs. 10.8 mg/g mycelia), equivalent to 2.6 g/L versus 1.6 g/L (Fig. 4E and S4B). However, the accumulation of the intermediate emodin also increased by 2.4-fold (Fig. 4E). Therefore, it is imperative to boost the transformation rate from emodin to physcion to further elevate the physcion titer.

### Enhancing 3-EOMT expression and SAM metabolism to optimize the conversion of Emodin to physcion

**Fig. 5:**
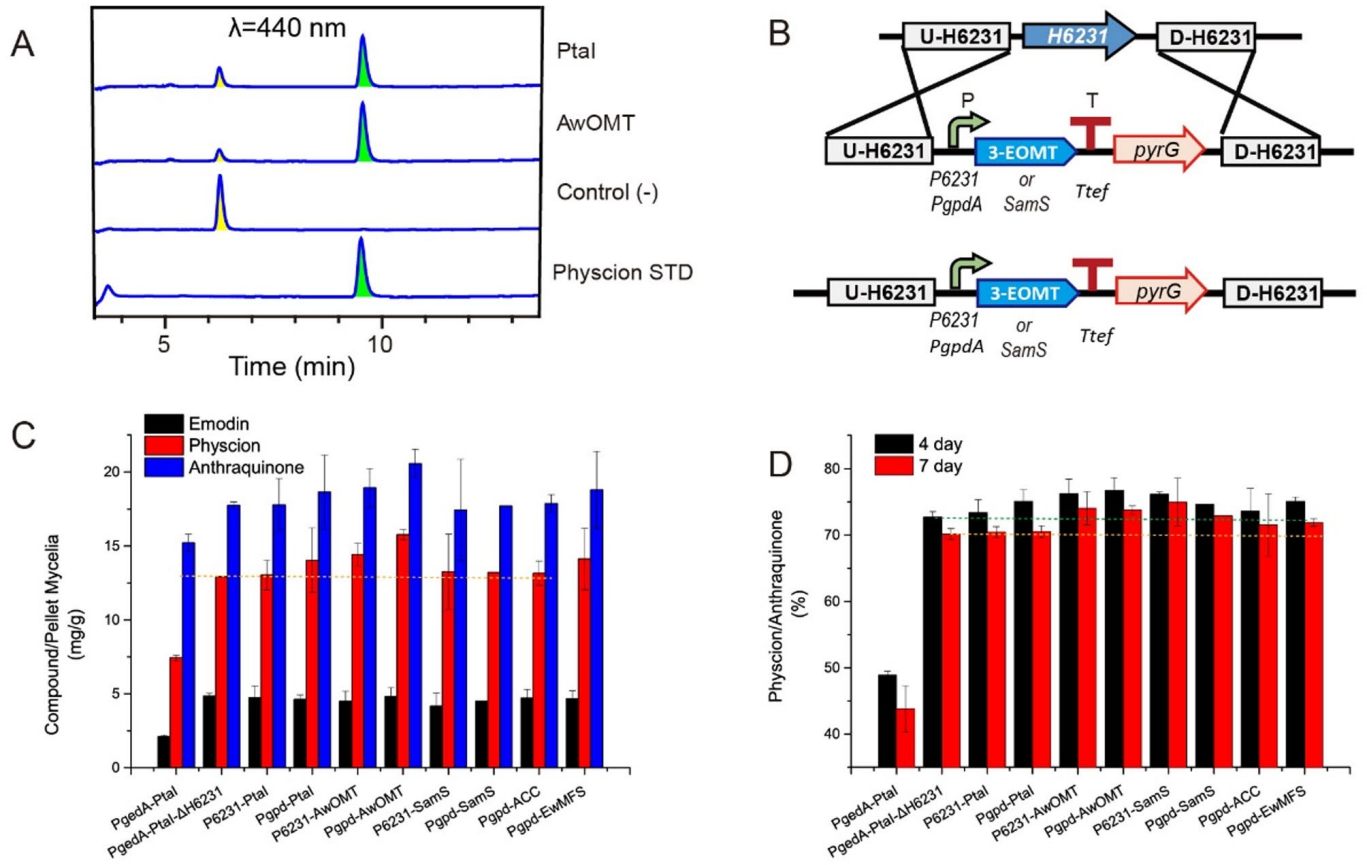
Enhancement of physcion production through 3-EOMT and SamS overexpression strategy. (**A**) HPLC analysis (λ = 440 nm) showing the enzymatic conversion by PtaI and AwOMT in the presence of SAM and emodin. Control (-) represents the negative control without enzyme. Physcion STD shows the standard reference. (**B**) Schematic representation of chromosomal integration of 3-EOMT or SamS at the *CYP-H6231* locus in the PgedA-PtaI-ΔpyrG strain. (**C**) Comparative analysis of emodin, physcion, and anthraquinone production titers (mg compound /g pellet mycelia) among different strains at day 4. Error bars indicate standard deviations from three independent replicates. (**D**) Comparison of emodin-to-physcion conversion rates (%) among different strains at day 4 and day 7. Error bars represent standard deviations from three independent replicates

To address the inefficient conversion of emodin to physcion, we hypothesized that the bottleneck may stem from either suboptimal expression of the 3-EOMT enzyme or limited availability of the methyl donor SAM. A candidate 3-EOMT from *Aspergillus wentii* (AwOMT) was identified, heterologously expressed in *E. coli* (pET28b), and purified via Ni-NTA chromatography (Fig. S5). Encouragingly, in vitro enzymatic reconstitution demonstrated that AwOMT has a higher catalytic efficiency than PtaI (88% vs. 72.7%), the enzyme used in the first-generation physcion-accumulating variant (Fig. 5A).

To enhance 3-EOMT expression, PtaI and AwOMT were individually overexpressed at the CYP-H6231 locus under the control of two strong constitutive promoters, *Pgpd* and *P6231*, creating four variants: Pgpd-PtaI, Pgpd-AwOMT, P6231-PtaI, and P6231-AwOMT (Table S2 and Fig. S6). These variants overexpressed 3-EOMT while deleting CYP-H6231 (Fig. 5B). Additionally, based on Yao et al. [15], the native SAM synthetase gene (*SamS*,* H6549*) was also overexpressed to generate two more variants: Pgpd-SamS and P6231-SamS (Table S2 and Fig. S6 and S7).

Despite these efforts, shake-flask fermentation revealed that most engineered strains exhibited only marginal improvements over PgedA-PtaI-ΔH6231. To facilitate consistent evaluation of biosynthetic performance across engineered strains, anthraquinone titers were primarily quantified in mg/g mycelia, which better reflects production efficiency by minimizing the impact of biomass variation. Specifically, Pgpd-AwOMT and P6231-AwOMT showed slight physcion increases at day four (~ 14–16 mg/g vs. 13 mg/g), while Pgpd-SamS and P6231-SamS displayed minor improvements at day seven (Fig. 5C and S8). Furthermore, the proportion of physcion within the total anthraquinones displayed only a marginal increase in the 3-EOMT and SamS overexpression variants compared to the PgedA-PtaI-ΔH6231 strain (around 71–75% versus 70% in the reference strain) (Fig. 5D). Additionally, supplementing SAM to the Pgpd-AwOMT culture did not lead to a notable improvement in physcion titer (Fig. S9), suggesting that SAM availability is unlikely to be the primary limiting factor. Likewise, attempts to increase precursor supply by overexpressing ACC [25], did not result in appreciable increases in physcion titer (Fig. 5C-D). Thus, we surmised that other limiting factors, such as toxicity resulting from intracellular compound accumulation or inefficient enzyme–substrate interaction, may play a more critical role, prompting us to explore further solutions.

### Exploring additional bottlenecks: compound export, protein fusion and co-expression strategy

To test this, a candidate emodin major facilitator superfamily (MFS) transporter EwMFS (GenBank: DAB41650.1) was identified from the emodin BGC in *Escovopsis weberi* [26] and overexpressed in the ΔgedA and PgedA-PtaI strains. Although EwMFS was predicted to contain multiple transmembrane regions (Fig. S10), its overexpression did not notably enhance product yields or facilitate compound export (Fig. 5C-D and S11), implying EwMFS lacks anthraquinone transport activity.

**Fig. 6:**
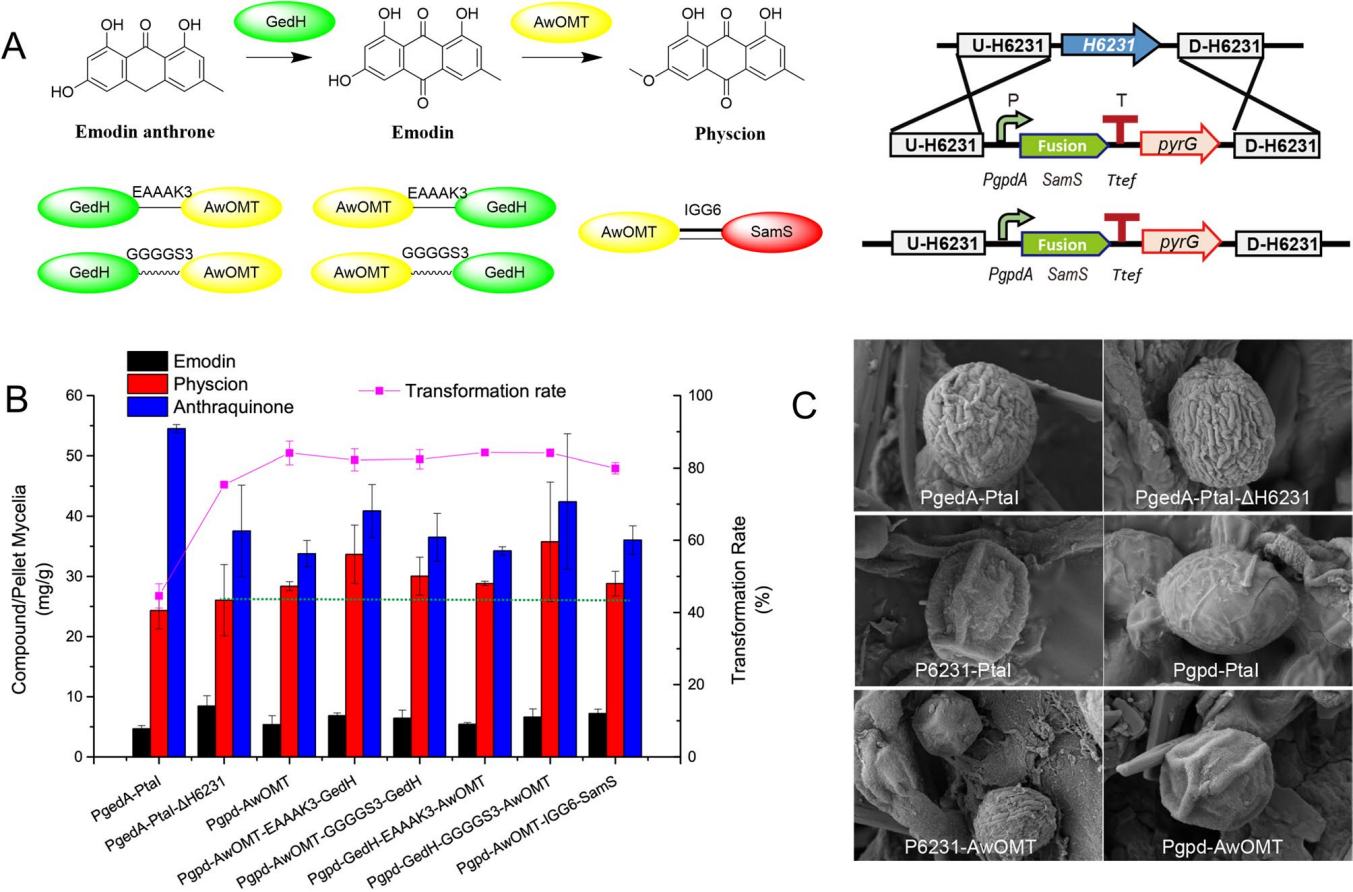
Optimization of physcion biosynthesis through protein fusion and co-expression strategies. (**A**) Left: Schematic representation of the emodin to physcion conversion pathway and various protein fusion combinations between GedH and AwOMT or GedH and SamS. Right: Diagram showing chromosomal integration strategy at the *CYP-H6231* locus in the PgedA-PtaI-ΔpyrG strain. (**B**) Quantitative analysis of metabolite production and conversion efficiency. Bar graph shows emodin, physcion, and anthraquinone titers (compound/pellet mycelia, mg/g), while the line graph indicates emodin-to-physcion transformation rates (%) for different fusion constructs at day 7. Error bars represent standard deviations from three independent replicates. (**C**) Cryo-scanning electron microscopy images showing morphological differences in a subset of spores among various genetic variants, including PgedA-PtaI, PgedA-PtaI-ΔH6231, P6231-PtaI, Pgpd-PtaI, P6231-AwOMT, and Pgpd-AwOMT strains

Next, a fusion protein strategy was employed to boost the conversion rate of newly generated emodin by 3-EOMT. The predicted anthrone oxygenase, GedH [27], was fused with the highly efficient 3-EOMT AwOMT using both rigid (EAAAK) and flexible (GGGGS) linkers to facilitate that newly synthesized emodin was efficiently utilized by 3-EOMT. In parallel, AwOMT was also co-overexpressed with SamS using the *IGG6* strategy [28], a 9-bp nucleotide sequence aiming at promote polycistronic gene expression in fungi (Fig. 6A and S12). Although all fusion protein variants demonstrated unsatisfactory physcion yields at day four because of apparently slow growth rate (Fig. S13), significant yield increases were observed over the subsequent three days, surpassing the CYP-deficient variant PgedA-PtaI-ΔH6231 (Fig. 6B). Notably, the best-performing strain Pgpd-GedH-GGGGS3-AwOMT showed approximately a 37% increase in physcion production at day 7 (36 mg/g vs. 26 mg/g) compared to the PgedA-PtaI-ΔH6231 strain. Furthermore, all mutants exhibited a higher emodin-to-physcion transformation rate (around 80–85%) compared to PgedA-PtaI-ΔH6231 (approximately 75%), indicating that this strategy, though only modestly successful, achieved the desired results.

### Morphological changes observed during strain engineering

During strain engineering, we observed a progressive decline in the growth capacity and spore viability of the variants, from the CYP-H6231 deletion strain PgedA-PtaI-ΔH6231 to the CYP-H6231 deletion and 3-EOMT overexpression strains (Pgpd-PtaI, Pgpd-AwOMT, P6231-PtaI, and P6231-AwOMT), and eventually to the protein fusion mutants. Additionally, we found that the spores of all variants, except for PgedA-PtaI and PgedA-PtaI-ΔH6231, exhibited altered spore morphology and blurred edges under the optical microscope.

This observed decline in spore morphology during strain modification prompted further cryo-scanning electron microscopy (cryo-SEM) analysis. Cryo-SEM revealed that the spores of both PgedA-PtaI and PgedA-PtaI-ΔH6231 displayed a rough surface with regular or irregular wrinkles. In contrast, in the CYP-H6231/3-EOMT co-engineered strains, some spores exhibited a smooth, wrinkle-free surface, while a significant number appeared oval-shaped (Fig. 6C and S14), which might be due to the intricate multistep preparation required for cryo-SEM. These findings indicate that the spores of those co-engineered variants exhibited greater fragility than the spores of the PgedA-PtaI and PgedA-PtaI-ΔH6231 strains.

These morphological changes, together with the modest titer improvement, suggest that overproduction of physcion imposes a significant metabolic burden. Thus, the CYP-H6231 is likely the most critical CYP enzyme in *A. terreus* responsible for detoxifying harmful intermediates during anthraquinone biosynthesis by hydroxylating emodin and physcion into more water-soluble, less toxic derivatives. Its deletion disrupts this detoxification route, leading to fragile mutants with compromised cell wall integrity, abnormal sporulation and impaired growth. Moreover, co-expression of 3-EOMT and SAM synthase exacerbated this burden, further affecting strain viability. These findings underscore the challenge of balancing pathway flux and host fitness in microbial cell factory design. Metabolic stress and insufficient detoxification are key limitations that must be addressed to achieve robust and scalable green biomanufacturing.

### Identification of potential substrate binding residues in CYP-H6231

**Fig. 7:**
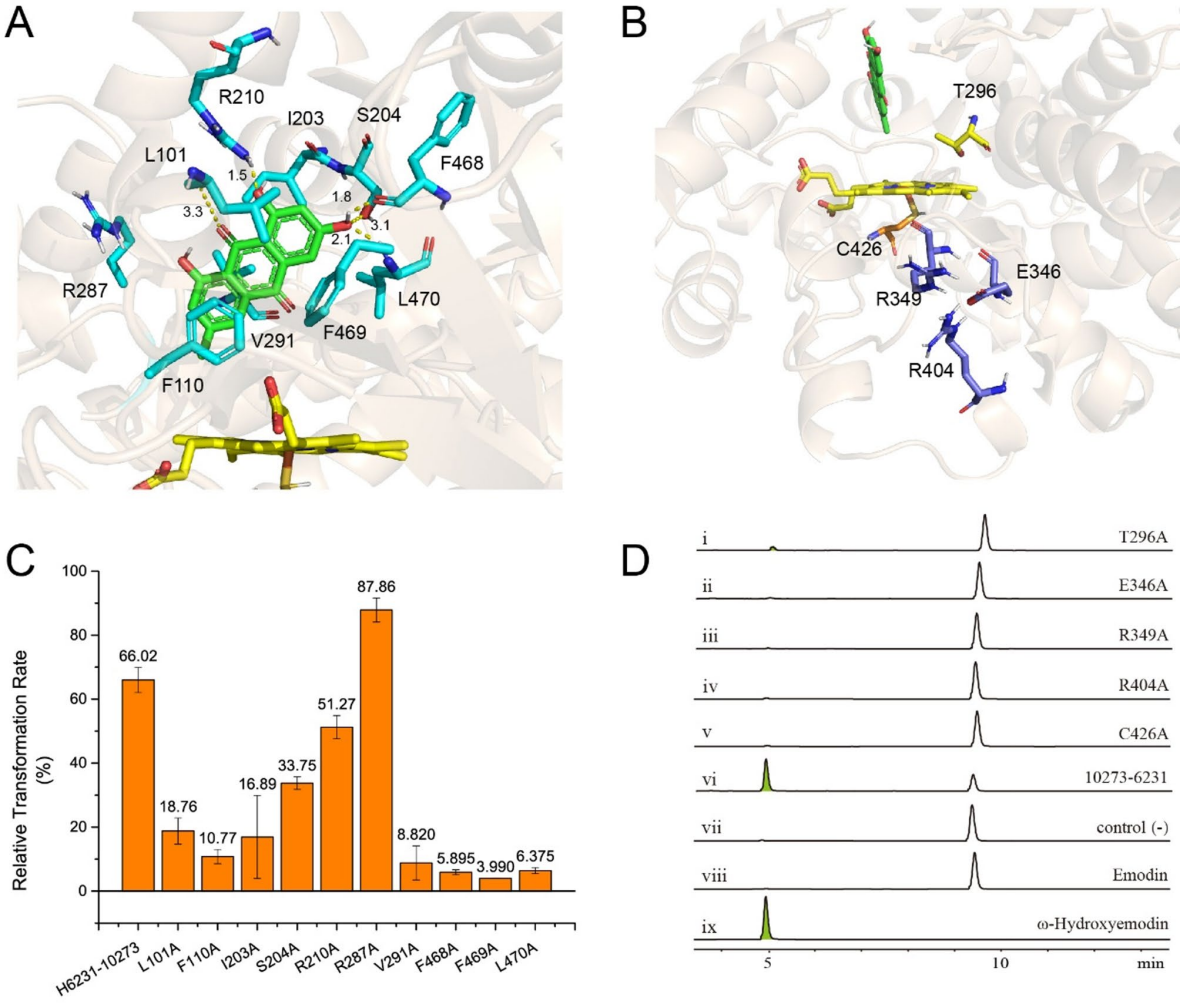
Structural analysis, site-directed mutagenesis, and enzymatic activity of CYP-H6231. (**A**) Close-up view of the emodin binding pocket. Emodin (green) is positioned near the heme group (yellow), with potential key substrate-binding residues shown in cyan and labeled. Numbers indicate interaction distances (in Å). **(B)** Catalytic site of CYP-H6231 highlighting key residues. Emodin (green sticks) and the heme group (yellow sticks) are shown, with essential catalytic residues colored blue (E–R–R triad), yellow (oxygen activation), and orange (heme binding), as labeled. (**C**) Comparison of the relative transformation rates of ω-hydroxyemodin by CYP-H6231 and its emodin-binding site mutants. Error bars represent standard deviations. (**D**) HPLC analysis of ω-hydroxyemodin production by CYP-H6231 catalytic site single-point mutants compared to wild-type CYP-H6231

To elucidate the mechanistic function of the key enzyme CYP-H6231, we employed AlphaFold2-based structural prediction, substrate docking, site-directed mutagenesis, and feeding assays. Because CYPs are typically membrane-bound, expressing them in *E. coli* poses challenges in functional characterization [24, 29]. As anticipated, attempts to heterologously express CYP-H6231 in *E. coli* using the pET28b vector were unsuccessful. To circumvent these limitations, the structural model of CYP-H6231 was predicted using AlphaFold2. Subsequently, emodin and the cofactor heme were docked into the predicted CYP-H6231 structure (Fig. S15). Based on AlphaFold2 predictions and substrate docking analyses, several key residues surrounding the docked emodin molecule —L101, F110, I203, S204, R210, R287, V291, F468, F469 and L470—were identified as potential substrate binding sites (Fig. 7A).

To validate the functional contributions of these residues, each was individually mutated to alanine and heterologously expressed in *S. cerevisiae* BY4742, along with CPR-H10273 to facilitate electron transfer [17] (Fig. S16). When emodin was fed to *S. cerevisiae* expressing these mutants, HPLC analysis revealed that the relative transformation rates of the L101A, F110A, I203A, V291A, F468A, F469A, and L470A variants were significantly reduced to 4.0%–18.8%, while the S204A and R210A mutants exhibited moderate reductions in transformation rate, retaining 33.8% and 51.3% activity, respectively (Fig. 7C and S17). These findings indicate that these residues play diverse roles in catalysis.

Structural analysis suggests that F110 and F469 engage in π–π stacking with the aromatic rings of emodin, whereas L101, I203, V291, F468, and L470 contribute hydrophobic interactions that help stabilize substrate binding. Additionally, L101, S204, R210, F468, and L470 are predicted to form hydrogen bonds with emodin. Together, these interactions are crucial for anchoring the substrate in the correct orientation within the binding pocket (Fig. 7A).

Further double and triple mutagenesis of L101, F468 and L470, three residues predicted to form hydrogen bonds with emodin, led to a near-complete loss of catalytic activity, with hydroxylated products nearly undetectable by HPLC (Fig. S16 and S17). This highlights their essential and synergistic contributions to substrate recognition and catalysis, consistent with the structural and single-mutant analysis.

### Functional analysis of conserved motifs and catalytic residues in CYP-H6231

To further identify crucial catalytic residues, a protein sequence alignment was performed between CYP-H6231 and multiple homologous CYP sequences, revealing four conserved regions characteristic of CYP enzymes [30–32]. The most prominent motif, FXXGXRXCXG (Domain D), represents the heme-binding domain, with C426 identified as critical for ligand binding to the heme. The EXXR (Domain B) and PER (Domain C) motifs form the E–R–R triad, which plays a pivotal role in stabilizing the heme pocket and maintaining the enzyme’s core structure. Additionally, the AGXDTT (Domain A) motif is involved in oxygen binding and activation (Fig. S18).

To validate the functional roles of these conserved residues, key positions including C426 (heme ligand binding), T296 (oxygen activation), and the E–R–R triad residues (E346, R349, R404) were individually mutated to alanine (Fig. 7B and S16). Feeding assays revealed that catalytic activity was completely abolished in the C426A, E346A, R349A, and R404A variants, and significantly reduced in the T296A mutant (Fig. 7D), demonstrating their essential roles in the catalytic function of CYP-H6231.

Together, these findings provide important mechanistic insights into CYP-H6231 function and lay the groundwork for rational enzyme engineering. In particular, targeted modifications at the substrate-binding interface may allow the design of CYP-H6231 variants that reduce hydroxylated byproduct formation while partially restoring detoxification capacity.

### Substrate specificity of CYP-H6231 and biosynthetic origin of fallacinol

To further explore the catalytic characteristics of CYP-H6231, its substrate specificity was assessed. Feeding of chrysophanol and physcion into the HXN301 culture, followed by LC-HRMS analysis, revealed that only trace amounts of aloe-emodin were produced, and no fallacinol was detected (Fig. S19). These results suggest that CYP-H6231 exhibits high substrate specificity, preferentially catalyzing the conversion of emodin to ω-hydroxyemodin. Importantly, the mechanistic understanding of CYP-H6231 obtained in this study provides a strategic basis for engineering the enzyme to construct a microbial cell factory capable of producing aloe-emodin from chrysophanol.

It was therefore hypothesized that the byproduct fallacinol is generated through sequential catalysis by CYP-H6231 and the 3-EOMT enzyme PtaI (Fig. S20A). To test this hypothesis, in vitro assays were performed using purified PtaI in the presence of ω-hydroxyemodin and SAM. The successful formation of fallacinol in these assays demonstrated that PtaI can utilize both emodin and its hydroxylated product, ω-hydroxyemodin, as substrates (Fig. S20B).

## Conclusion

Through systematic metabolic engineering, we established two distinct *A. terreus* cell factories for high-yield, high-purity production of physcion and emodin, respectively. While CYP-H6231 deletion proved the most effective strategy for optimizing physcion biosynthesis, it also led to substantial emodin accumulation. Comprehensive characterization revealed that neither 3-EOMT expression levels nor SAM availability primarily constrain emodin-to-physcion conversion, as overexpression and fusion strategies yielded only modest improvements. We successfully identified and elucidated the enzymatic mechanism of CYP-H6231, a crucial detoxification enzyme and its dedicated CPR, providing mechanistic insights into anthraquinone hydroxylation. This work establishes improved and scalable microbial platforms for the production of both physcion and emodin, along with a rational framework for engineering anthraquinone biosynthesis, offering a sustainable alternative to plant-based extraction.

## Supplementary Information

Supplementary Material 1.

## Data Availability

No datasets were generated or analysed during the current study.
