# Supplementary material for: Multi-layered engineering of Aspergillus terreus enhances biosynthesis of the plant-derived fungicide physcion
Source: Microb Cell Fact. 2025 Nov 13;24:234. doi: 10.1186/s12934-025-02857-z (PMC12613595; doi:10.1186/s12934-025-02857-z)
Supplement: Supplementary file 1 — Supplementary Material 1. [file 12934_2025_2857_MOESM1_ESM.docx]

**Supporting Information**

**Multi-Layered Engineering of *Aspergillus terreus* Enhances Biosynthesis of the Plant-Derived Fungicide Physcion**

Zilin Ren^1,2,3,4,#^, Yingying Xue^1,2,3,4,#^, Ning Xu^1,2,3,4,5^, Dandan Feng^1,2,3,4^, Ce Geng^1,2,3,4^, Yongsong Wu^1,2,3,4,6^, Dan Liu^1,2,3,4^, Linshui Zhao^1,2,3,4^, Xiaoxi Zhang^1,2,3,4^, Honglei Ma^1,2,3,4,7^, Xuenian Huang^1,2,3,4,5,7^, Feifei Qi^1,2,3,4,5,7,^*, Xuefeng Lu^1,2,3,4,7,8,^*

^1^ Shandong Provincial Key Laboratory of Synthetic Biology, Qingdao Institute of Bioenergy and Bioprocess Technology, Chinese Academy of Sciences, Qingdao, Shandong 266101, China

^2^ Key Laboratory of Biofuels, Qingdao Institute of Bioenergy and Bioprocess Technology, Chinese Academy of Sciences, Qingdao, Shandong 266101, China

^3^ Shandong Energy Institute, Qingdao, Shandong 266101, China

^4^ Qingdao New Energy Shandong Laboratory, Qingdao, Shandong 266101, China

^5^ School of Biological Science and Technology, University of Jinan, Jinan, Shandong 250022, China

^6^ College of Life Science, Dalian Minzu University, Dalian, Liaoning 116600, China

^7^ University of Chinese Academy of Sciences, Beijing 100049, China

^8^ Marine Biology and Biotechnology Laboratory, Qingdao National Laboratory for Marine Science and Technology, Qingdao, Shandong 266237, China

^#^ These authors contributed equally to this work.

*** Corresponding author.** Qingdao Institute of Bioenergy and Bioprocess Technology, Chinese Academy of Sciences, No. 189 Songling Road, Qingdao 266101, China.

**E-mail addresses:** lvxf@qibebt.ac.cn (X. Lu), qiff@qibebt.ac.cn (F. Qi).

**TABLE OF CONTENTS**

[Spectral data of compounds 3](#_Toc205409314)

[Supporting Tables 4](#_Toc205409315)

[Table S1 DNA and protein sequences of CYP-H6231 and CPR-H10273 4](#_Toc205409316)

[Table S2. Strains constructed and used in this study 8](#_Toc205409317)

[Table S3. Oligonucleotide primers used in this study 9](#_Toc205409318)

[Table S4. The FPKM of cytochrome P450s in HXN301 and PgedA-PtaI 13](#_Toc205409319)

[Table S5. The CPRome of *Aspergillus terreus* 14](#_Toc205409320)

[Supporting Figures 15](#_Toc205409321)

[Fig. S1 LC-HRMS characterization of ω-hydroxyemodin, fallacinol, emodin, and physcion isolated from Pgpd-PtaI fermentation culture 15](#_Toc205409322)

[Fig. S2 HPLC analysis of emodin feeding assay in HXN301 16](#_Toc205409323)

[Fig. S3 PCR verification of the ΔgedA-ΔH6231, ΔgedA-ΔH2247, ΔgedA-ΔH3969 and PgedA-PtaI-ΔH6231 17](#_Toc205409324)

[Fig. S4 Production titers (g/L) of anthraquinone compounds by different genetic variants 18](#_Toc205409325)

[Fig. S5 SDS-PAGE analysis of purification of AwOMT 19](#_Toc205409326)

[Fig. S6 PCR verification of the 3-EOMT, SamS, acetyl-CoA carboxylase (ACC) and EwMFS overexpression variants 21](#_Toc205409327)

[Fig. S7 Biosynthetic pathway of SAM in *A. terreus* and transcription levels of relevant genes 22](#_Toc205409328)

[Fig. S8 Emodin, physcion and anthraquinone production by different genetic variants on day 7 23](#_Toc205409329)

[Fig. S9 Time course analysis of emodin (A) and physcion (B) production after feeding different SAM concentrations 24](#_Toc205409330)

[Fig. S10 Transmembrane domain prediction analysis of the EwMFS transporter protein using TMHMM 2.0 25](#_Toc205409331)

[Fig. S11 Analysis of metabolite production and compound distribution in strains expressing the EwMFS transporter 26](#_Toc205409332)

[Fig. S12 PCR verification of the protein fusion and co-expression variants 27](#_Toc205409333)

[Fig. S13 Analysis of emodin, physcion, and anthraquinone production levels and emodin-to-physcion transformation efficiency in different variants 28](#_Toc205409334)

[Fig. S14 Cryo-scanning electron microscopy images showing spore morphology changes in P6231-PtaI, Pgpd-PtaI, and P6231-AwOMT strains 29](#_Toc205409335)

[Fig. S15 Overall structure of CYP-H6231 enzyme with docked emodin in the active site 30](#_Toc205409336)

[Fig. S16 PCR verification of CYP-H6231 mutants expressed in *Saccharomyces cerevisiae* 31](#_Toc205409337)

[Fig. S17 HPLC analysis of ω-hydroxyemodin production in *S. cerevisiae* strains expressing site-directed mutants of CYP-H6231 32](#_Toc205409338)

[Fig. S18 Multiple protein sequence alignment between CYP-H6231 and its analogous enzymes 33](#_Toc205409339)

[Fig. S19 LC-HRMS analysis of chrysophanol feeding assays in the HXN301 culture 35](#_Toc205409340)

[Fig. S20 Hypothetical biosynthesis pathway of the hydroxylation product fallacinol 36](#_Toc205409341)

[Supporting References 37](#_Toc205409342)

# Spectral data of compounds

Physcion, yellow amorphous powder, HRESIMS *m/z* calculated for C_16_H_13_O_5_ 285.0757, experimental 285.0761 [M+H]^-^. ^1^H-NMR (DMSO-*d*_6_, 600 MHz) *δ* 2.44 (3H, s), 3.94 (3H, s), 6.90 (1H, d, *J* = 2.4 Hz), 7.21 (1H, d, *J* = 2.4 Hz), 7.22 (1H, s), 7.55 (1H, s), 11.98 (1H, s), 12.19 (1H, s); ^13^C-NMR (DMSO-*d*_6_, 150 MHz) *δ* 21.5, 56.4, 106.6, 107.7, 109.9, 113.5, 120.6, 124.3, 132.9, 134.9, 148.6, 161.5, 164.4, 166.2, 181.3, 190.0. ^1^H and ^13^C NMR data were in good agreement with the published data [1].

Emodin, yellow amorphous powder, HRESIMS *m/z* calculated for C_15_H_9_O_5_ 269.0455, experimental 269.0460 [M-H]^-^. ^1^H NMR (acetone-*d_6_*, 600 MHz) *δ* 2.45 (3H, s), 6.66 (1H, s), 7.11 (1H, s), 7.24 (1H, s), 7.53 (1H, s), 12.06 (1H, s), 12.16 (1H, s); ^13^C NMR (acetone-*d*_6_, 150 MHz) *δ* 22.0, 108.9, 109.9, 110.3, 114.5, 121.5, 124.9, 134.3, 136.6, 149.5, 163.3, 166.3, 166.8, 182.2, 191.7. ^1^H and ^13^C NMR data were in good agreement with the published data [2].

ω-Hydroxyemodin, yellow amorphous powder, HRESIMS calculated for C_15_H_9_O_6_ 285.0405, experimental 285.0406 [M-H]^-^. ^1^H NMR (DMSO-*d*_6_, 600 MHz) *δ* 4.60 (2H, s), 6.59 (1H, s), 7.11 (1H, s), 7.23 (1H, s), 7.61 (1H, s); ^13^C NMR (DMSO-*d*_6_, 150 MHz) *δ* 62.5, 108.4, 109.3, 109.4, 114.5, 117.6, 121.3, 133.4, 135.6, 153.3, 162.0, 165.0, 166.2, 181.8, 190.2. ^1^H and ^13^C NMR data were in good agreement with the published data^3^.

Fallacinol, yellow needle crystal, HRESIMS calculated for C_16_H_13_O_6_ 301.0707, experimental 301.0703 [M+H]^-^. ^1^H NMR (DMSO-*d*_6_, 600 MHz) *δ* 3.92(3H, s), 4.60(2H, s), 6.85(1H, s), 7.15(1H, s), 7.25(1H, s), 7.64(1H, s), 11.99(1H, s), 12.15(1H, s); ^13^C NMR (DMSO-*d*_6_, 150 MHz) *δ* 56.4, 61.9, 106.5, 107.5, 109.8, 114.0, 117.1, 120.8, 132.8, 134.7, 153.0, 161.4, 164.3, 166.0, 181.1, 189.9. ^1^H and ^13^C NMR data were in good agreement with the published data [3].

# Supporting Tables

## Table S1 DNA and protein sequences of CYP-H6231 and CPR-H10273

| **CYP-H6231 DNA sequence**  ATGGGTTTTCTAACGCTCTTCCAGTCCATTTCTCTTGTTCTCTTTCTAGTGAGTTATCCAGCTCGACCTATTCTGCGATTACCTGCTGAGCACCATACTAGAGACTGCTATATCGGAGATTCAACGGCCCCCTTGCACATCTTCCCGGCCCCGAGATCTCCAGATGGACAGGCCTCCTCAGTACGATCTACTGGTTCCGCGGGCAGAAGCCCAACTACGTCCACTATCTCCATGAGAAATACGGTATGCGAGCGACAGCCGTCAAGGCGTACCATGCTAGCTACTAATGCCCCCGGATAAAGGCCCCATAGTCCGCGTCACCCCCGAAGAAGTCGACATCTGCGATATCACCGCCGCAAAGGAAATCCACAAGACGGGCGGCCGGTTCCTCAAGTCCAACTTCTACCATGCGTTGGCGCCGCCCAACACGGAGAGCATCTTCTCCACGACCGATCCGGCCTTCCATTCCGCACATCGGCGTCTCCTCGCCACGCCCATCTCGGATTCGTCGCTGACGGGCTTTGAGCAGGTTATCGCGGGGAAAGTGCACCTGGCGGTGCGCCGGATGGGTGAGGAGATGCGCTCCCGCGGCGCGATGGACGTCTTCAAGTGGTGGCTGTTCATGGCCACGGACGTCATCGGGGAGCTGAGCTTTGGTGAGTCCTTTCGTATGCTGGAGTCTGGGCAGGTAAGACACACATACACACACAACAGCTCCGGGCCAGCTGATCGCATCCGTCGCATAGAAAAACCAATACATCCTCGACCTCGAGCAGATATCCTCGTTGGCACCGGTGCGCACGACGTTCCCGTCGCTCGTGCGGCTCGGCTCCCTCCTGCCTCTGCCTGTCTTCCAGCGGGTCGCGGCGGCGGGCCAGCGGCTCATCGACTACGCGCAGCAGTCGATCGACCGGTACGCCCGTCTCGTGGAGCGGTCGGGGGTGAGCGCGCCGCCGCCGACGCTGTTCACGAAGCTGTACAACGCGGGCAAGGATGGCCTGTCTACGACGGATATCCGGAACGAAGCGCGGGCGTACATCGTTGCCGGGAGCGACACCACCGCAATCAGTTTGACGTATCTTGTCTATGCGGTGTGTCGGGACGAGAGGGTCCACGCGAGGCTTGTCGATGAGGTGGCCGCTCTGCCTGAGGATTTTGACGATAGGATGATGCGCGAGCTGCCCTATCTTAACTGGGTGATTAACGAGGCTTTGCGATTGTATACGGCCGTCCCGTTTGGTCTGCCGAGGGCTGTTCCGGCTGAGGGAGCCGAGTTTTTGGGATACCGGCTTCCTGGCGGGGTGATCGTTTCAACGCAATCGTACAGTCTTCATCGGGATGGAGAGATATTTCCCGAGCCGGACAGGTGAGTGTTCGTCCTGTTGTCTGAGTCTGTAGCTGGTTGCTCATGGAACAAGATTCTACCCGGAGCGCTGGGAAACGGTCACCAAGGAAATGAAAGAAGCGTCTATGCCGTTCGGGAAAGGGGCACGCGGTATGTATCCTCCAGGCGCTTGGGACCTAGACGTTGTCTCATTGTATGCTGTCTCTAGCTTGTATTGGGATCCATCTAGCCCGTAGGGAACTGCGTCTGGCAACGGTGCTGTTCTTCCGCGCTTTCCCGCAAGCAAGGATATCGACTAGAGAAGGGATGGGCGAGGACGATATGGAGATGAAGAGCTTCTTTCTTATGGCGCCCAAGGGGCATCGCTGCCTTATAGAGGCGTAG |
| --- |
| **CYP-H6231 Protein sequence**  MGFLTLFQSISLVLFLRLLYRRFNGPLAHLPGPEISRWTGLLSTIYWFRGQKPNYVHYLHEKYGPIVRVTPEEVDICDITAAKEIHKTGGRFLKSNFYHALAPPNTESIFSTTDPAFHSAHRRLLATPISDSSLTGFEQVIAGKVHLAVRRMGEEMRSRGAMDVFKWWLFMATDVIGELSFGESFRMLESGQKNQYILDLEQISSLAPVRTTFPSLVRLGSLLPLPVFQRVAAAGQRLIDYAQQSIDRYARLVERSGVSAPPPTLFTKLYNAGKDGLSTTDIRNEARAYIVAGSDTTAISLTYLVYAVCRDERVHARLVDEVAALPEDFDDRMMRELPYLNWVINEALRLYTAVPFGLPRAVPAEGAEFLGYRLPGGVIVSTQSYSLHRDGEIFPEPDRFYPERWETVTKEMKEASMPFGKGARACIGIHLARRELRLATVLFFRAFPQARISTREGMGEDDMEMKSFFLMAPKGHRCLIEA |
| **CPR-H10273 DNA sequence**  ATGGCTCAACTCGACACTCTCGACCTGGTGGTCCTGGTGGTGCTTTTGGTGGGTAGCGCCGCCTACTTCACCAAGGGCACCTACTGGGCCGTTCCCAAGGACCCGTATGCCGCCTCCGGTCCCGCCATGAATGGTGGTGCCAAGGCGGGCAAATCCAGGGACATCATTGAGAAAATGGAAGAGACTGGCAAGAACTGTGTGATTTTCTACGGCTCGCAGACCGGTACCGCCGAGGATTATGCGTCGCGCCTGGCCAAGGAAGGCTCCCAGCGTTTCGGCCTCAAGACCATGGTCGCAGATCTGGAAGACTACGATTATGAGAACCTGGACAAGTTCCCCGAGGACAAGGTTGCCTTCTTCGTCATGGCCACCTATGGTGAGGGTGAACCCACCGACAACGCCGTCGAGTTCTACCAGTTCATCTCGGGTGAGGACGTCGCGTTCGAGAGCGGCGCCTCCGCCGACGACAAGCCCCTGTCCTCCCTCAAGTATGTCACTTTCGGTCTCGGTAACAACACCTATGAGCACTATCAGGCTATGGTTCGCAATCTGGATGCCGCTCTCACCAAGCTGGGTGCGCAGCGCATTGGAGATGCTGGTGAAGGCGATGACGGCGCTGGCACCATGGAAGAAGATTTCCTGGCCTGGAAAGAGCCCATGTGGACCGCCCTGTCCGAGGCCATGAACCTTCAGGAGCGCGAGGCCGTCTATGAACCGGTGTTCTCGGTCACGGAAGATGAATCCCTGTCCCCCGAAGACGAGGCCGTCTACCTCGGTGAGCCGACCAAGGGTCATCGTGACGGCACCCCCAGTGGCCCGTATTCCGCTCACAACCCCTTCATCGCCCCCATCGTCGAGTCTCGTGAACTATTCAACGTCAAGGACCGTAACTGTCTGCACATGGAGATCAGCATCGCTGGTAGCAACCTTTCTTACCAGACTGGTGATCACATCGCGATTTGGCCCACGAACGCTGGTGCCGAGGTGGACCGGTTCCTCCAGGTGTTTGGTCTTGAGAACAAGCGTCATTCCGTCATCAACGTCAAGGGTATCGATGTGACCGCCAAGGTTCCCATTCCGACTCCCACCACTTATGATGCTGCTGTTCGCTACTACATGGAAATCGCTGCGCCCGTCTCCCGTCAGTTTGTGGCTACCCTGGCTGCGTTTGCTCCCGATGAGGAGACCAAGGCGGAAATCGTGCGTTTGGGTAGCGACAAGGACTACTTCCACGAGAAAATCAGCAACCAGTGCTTCACCATCGCTCAGGCTCTTCAGAGTGTCACCTCCAAGCCCTTCTCGGCTGTCCCGTTCTCTCTGCTTATCGAGGGTCTCAATAAGCTCCAGCCCCGTTACTACTCCATCTCTTCCTCCTCCATGGTCCAGAAGGATAAGATCAGCATTACTGCCGTCGTGGAATCCACTCGCTTGCCTGGTGCCGCCCACCTTGTCAAGGGTGTCACGACCAACTATCTCCTTGCCCTGAAGCAAAAGCAGAATGGCGATCCGTCTCCCGACCCTCACGGCTTAACTTATACTATCACTGGGCCCCGTAACAAGTACGACGGAATCCACGTTCCCGTTCACGTCCGCCACTCCAATTTCAAGCTCCCCTCTGATCCCTCTCGGCCCATTATCATGGTTGGCCCTGGTACCGGTGTGGCTCCCTTCCGCGGATTCATCCAGGAGCGTGCCGCCTTGGCCGCCAAGGGTGAGAACGTCGGTCCCACCGTGTTGTTCTTTGGATGCCGCAAGCGCGATGAGGACTTTATGTACGCAGATGAATTCAAGACCTACCAGGAACAGCTTGGGGACAAACTTCAGATCATTACTGCGTTTTCTCGTGAAACCTCCCAGAAGGTGTATGTTCAGCACAGACTGCGTGAACACTCCGATCTGGTGAGCAGCCTCCTGAAGCAGAAGGCTAACTTTTACGTCTGCGGTGACGCCGCCAACATGGCGCGTGAAGTCAACCTTGTGCTTGGCCAGATCATCGCGCAACAGCGTGGTCTCCCGGCTGAACGGGCCGAGGAAATGGTGAAGCACATGCGCAGCAGCGGCAGCTACCAGGAGGACGTGTGGTCATGA |
| **CPR-H10273 Protein sequence**  MAQLDTLDLVVLVVLLVGSAAYFTKGTYWAVPKDPYAASGPAMNGGAKAGKSRDIIEKMEETGKNCVIFYGSQTGTAEDYASRLAKEGSQRFGLKTMVADLEDYDYENLDKFPEDKVAFFVMATYGEGEPTDNAVEFYQFISGEDVAFESGASADDKPLSSLKYVTFGLGNNTYEHYQAMVRNLDAALTKLGAQRIGDAGEGDDGAGTMEEDFLAWKEPMWTALSEAMNLQEREAVYEPVFSVTEDESLSPEDEAVYLGEPTKGHRDGTPSGPYSAHNPFIAPIVESRELFNVKDRNCLHMEISIAGSNLSYQTGDHIAIWPTNAGAEVDRFLQVFGLENKRHSVINVKGIDVTAKVPIPTPTTYDAAVRYYMEIAAPVSRQFVATLAAFAPDEETKAEIVRLGSDKDYFHEKISNQCFTIAQALQSVTSKPFSAVPFSLLIEGLNKLQPRYYSISSSSMVQKDKISITAVVESTRLPGAAHLVKGVTTNYLLALKQKQNGDPSPDPHGLTYTITGPRNKYDGIHVPVHVRHSNFKLPSDPSRPIIMVGPGTGVAPFRGFIQERAALAAKGENVGPTVLFFGCRKRDEDFMYADEFKTYQEQLGDKLQIITAFSRETSQKVYVQHRLREHSDLVSSLLKQKANFYVCGDAANMAREVNLVLGQIIAQQRGLPAERAEEMVKHMRSSGSYQEDVWS |

## Table S2. Strains constructed and used in this study

| *A. terreus* strains | Genotype or characteristics | Reference |
| --- | --- | --- |
| HXN301 | Statins-producing strain | Our lab |
| ∆gedA | HXN301-*∆pyrG*, *ku80:: pyrG_An_::gedR,* ∆*gedA* | [4] |
| P*gedA*-PtaI | HXN301-*∆pyrG*, *ku80:: pyrG_An_::gedR,* *gedA:: ptrA::PtaI* | [4] |
| ΔgedA-ΔpyrG | HXN301-*∆pyrG*, *ku80:: pyrG_An_::gedR,* ∆*gedA,* ∆*pyrG* | This study |
| ΔgedA-ΔH10273 | Δ*gedA*-Δ*pyrG,* ∆H*10273* | This study |
| ΔgedA-ΔH6231 | Δ*gedA*-Δ*pyrG,* ∆*H6231* | This study |
| ΔgedA-ΔH2247 | Δ*gedA*-Δ*pyrG,* ∆*H2247* | This study |
| ΔgedA-ΔH3979 | Δ*gedA*-Δ*pyrG,* ∆*H3979* | This study |
| ΔgedA-EwMFS | Δ*gedA*-Δ*pyrG, H6231:: pyrG_An_::EwMFS* | This study |
| PgedA-PtaI-ΔpyrG | HXN301-*∆pyrG*, *ku80:: pyrG_An_::gedR,* *gedA:: ptrA::PtaI,* ∆*pyrG* | This study |
| PgedA-PtaI-ΔH6231 | P*gedA*-*PtaI*-Δ*pyrG,* ∆*H6231* | This study |
| Pgpd-PtaI | P*gedA*-*PtaI*-Δ*pyrG, H6231:: pyrG_An_::PtaI* | This study |
| Pgpd-AwOMT | P*gedA*-*PtaI*-Δ*pyrG, H6231:: pyrG_An_::AwOMT* | This study |
| P6231-PtaI | P*gedA*-*PtaI*-Δ*pyrG, H6231:: pyrG_An_::PtaI* | This study |
| P6231-AwOMT | P*gedA*-*PtaI*-Δ*pyrG, H6231:: pyrG_An_::AwOMT* | This study |
| Pgpd-SamS | P*gedA*-*PtaI*-Δ*pyrG, H6231:: pyrG_An_::SamS* | This study |
| P6231-SamS | P*gedA*-*PtaI*-Δ*pyrG, H6231:: pyrG_An_::SamS* | This study |
| Pgpd-EwMFS | P*gedA*-*PtaI*-Δ*pyrG,* *H6231:: pyrG_An_::EwMFS* | This study |
| Pgpd-ACC | P*gedA*-*PtaI*-Δ*pyrG, H6231:: pyrG_An_::ACC* | This study |
| Pgpd-AwOMT-EAAAK3-GedH | P*gedA*-*PtaI*-Δ*pyrG, H6231:: pyrG_An_::AwOMT-EAAAK3-GedH* | This study |
| Pgpd-AwOMT-GGGGS3-GedH | P*gedA*-*PtaI*-Δ*pyrG, H6231:: pyrG_An_::AwOMT-GGGGS3-GedH* | This study |
| Pgpd-GedH -EAAAK3-AwOMT | P*gedA*-*PtaI*-Δ*pyrG, H6231:: pyrG_An_::GedH -EAAAK3-AwOMT* | This study |
| Pgpd-GedH -GGGGS3-AwOMT | P*gedA*-*PtaI*-Δ*pyrG, H6231:: pyrG_An_::GedH -GGGGS3-AwOMT* | This study |
| Pgpd-AwOMT-IGG6-SamS | P*gedA*-*PtaI*-Δ*pyrG, H6231:: pyrG_An_::AwOMT-IGG6-SamS* | This study |

## Table S3. Oligonucleotide primers used in this study

| **Primer** | **Sequence (5' - 3')** |
| --- | --- |
| UpyrGAn-F | TCCAGAGGCTCTGAGTTTGC |
| UpyrGAn-R | GAGATGGTGATTGAGCTAGTTTACACTCTGGGAGGATCCA |
| DpyrGAn-F | TGGATCCTCCCAGAGTGTAACTAGCTCAATCACCATCTC |
| DpyrGAn-R | GGCAGAGCTGACTGGAGAGA |
| CpyrGAn-F | GATGGCAGTCGACATCGGAT |
| CpyrGAn-R | GTCAACGTCAGTCTATCGAG |
| Uan-F | CTCCGAACACCCACAATAGAGCATG |
| Dan-R | CTCCGAACACCCACAATAGAGCATG |
| U10273-F | ATAGCAGGGGCTCTTCCTCTC |
| U10273-R | CCATTGGAGCTCGTATTCCCTGCTGGCTCCATGAACTTTCTTTCGCG |
| D10273-F | TCAATGCGGTACGACGATTTGATGCAATTGCGTCCGTGGAGAGGTG |
| D10273-R | GAAGAAGTACTCCAGTTGCCAGC |
| U6231-F | CACACATGGGGTTCGATGTTTCG |
| U6231-R | ACTTCGTATAGCATACATTATACGAAGTTATGGCTGGCAGTTCAATCTGGGGAC |
| D6231-F | TTCGTATAATGTATGCTATACGAAGTTATGGCGTAGTTGTTAGTATTGGATATG |
| D6231-R | TACTGGAAGAAATACCTGGCCGGC |
| U2247-F | AATTTCAATGGGCTACTCTGCAG |
| U2247-R | CCATTGGAGCTCGTATTCCCTGCTGGATCGTGGGCTTGGTTGAAATG |
| D2247-F | TCAATGCGGTACGACGATTTGATGCTTGGGCATATACGCGGGAGTTTG |
| D2247-R | TGATGTTATGCTTGTGTACCTGGAG |
| U3979-F | AGACCTTTTTGTTGGTCCGGACTC |
| U3979-R | CATTGGAGCTCGTATTCCCTGCTGCTCACAGATCCTGGGTTCCATTGAC |
| D3979-F | CAATGCGGTACGACGATTTGATGCCGCGTCTACTTACTCGGTAGATCTC |
| D3979-R | AGCTAGTCGGGCTAGCTTGTAC |
| C10273-F | ATGACAGTGTGCAGTAGGTGTAG |
| C10273-R | GTACCGTCCTCGGAGATGTTAC |
| C6231-F | GAATTAAGGCTGCACCGGCG |
| C6231-R | AGACACGCCCAAGAAGCAAAC |
| C2247-F | ACCAAAAGCCCATGAGTACTGTCG |
| C2247-R | TGGCATCCCAATAGCCTGGACTC |
| C3979-F | TGTTGGTCCGGACTCTTACGC |
| C3979-R | TACGAGGATTTCCATTGCGTGGC |
| pyrGAn-F | CAGCAGGGAATACGAGCTCCAATG |
| pyrGAn-R | GCATCAAATCGTCGTACCGCATTG |
| pyrG-in-R | TCGACTGGTGGACCCTGATTC |
| pyrG-in-F | TTGGTCAGCAGTACCAGACGC |
| *PgpdAt*-6231-F | TAGTCCCCAGATTGAACTGCCAGCCCCCTTGTATCTCTACACACAGGC |
| *PgpdAt*-R | TGATGTCTGCTCAAGCGGGGTAG |
| Ttef-F | GCGGACATTCGATTTATGCCGTTAT |
| Ttef-R | ACTTCGTATAGCATACATTATACGAAGTTATGTATTGGGATGAATTTTGTATGCACG |
| pyrGAn-loxP-F | CAATACATAACTTCGTATAATGTATGCTATACGAAGTTATCAGCAGGGAATACGAGCTC |
| pyrGAn-loxP-R | ACGCCATAACTTCGTATAGCATACATTATACGAAGTTATGCATCAAATCGTCGTACCG |
| P6231-PtaI-F | TAGTCCCCAGATTGAACTGCCAGCCATGCCCTCCCCCACCAATC |
| *PgpdAt*-PtaI-F | AGCTACCCCGCTTGAGCAGACATCAATGCCCTCCCCCACCAATC |
| Ttef-PtaI-R | ATAACGGCATAAATCGAATGTCCGCTCAGTGGTGGTGGTGGTGGTGC |
| P6231-AwOMT-F | TAGTCCCCAGATTGAACTGCCAGCCATGGCCTCCCCCTTGACCTTG |
| *PgpdAt*-AwOMT-F | AGCTACCCCGCTTGAGCAGACATCAATGGCCTCCCCCTTGACCTTG |
| Ttef-AwOMT-R | ATAACGGCATAAATCGAATGTCCGCTCAGTGGTGGTGGTGGTGGTG |
| AwOMT-F | GCCTCCCCCTTGACCTTGTTGG |
| AwOMT-R | GACGTCCAGCTGGGCTTCAATG |
| P6231-SamS-F | TAGTCCCCAGATTGAACTGCCAGCCATGGGCAGCGTCGCTGAGCCTAAG |
| *PgpdAt*-SamS-F | AGCTACCCCGCTTGAGCAGACATCAATGGGCAGCGTCGCTGAGCCTAAG |
| SamS-R | ATAACGGCATAAATCGAATGTCCGCTTAGAACTTGAGGGCCTTGGGCTT |
| *PgpdAt*-ACC-F | AGCTACCCCGCTTGAGCAGACATCAATGGCCTCCACCAATGGTCAC |
| *PgpdAt*-ACC-R | ATAACGGCATAAATCGAATGTCCGCTTAAGCAGATCCCAAGTACTTGAGG |
| *PgpdAt*-EWMFS-F | GCTACCCCGCTTGAGCAGACATCAATGCTGTCCAAAAAGCCCGCCGGTG |
| *PgpdAt*-EWMFS-R | ATAACGGCATAAATCGAATGTCCGCTTAGGACTTGACGTCGGTGGTTTC |
| pyrGAn-in-R | TCGACTGGTGGACCCTGATT |
| H6231-in-F | ATGGGTTTTCTAACGCTCTTCCAG |
| H6231-in-R | CTACGCCTCTATAAGGCAGCG |
| pyrGAn-in-F | TTGGTCAGCAGTACCAGACGC |
| AwOMT-IGG6-  SamS-F | CTTCATTGAAGCCCAGCTGGACGTCCAATCAAACATGGGCAGCGTCGCTGAG |
| *Ttef*-SamS-R | ATAACGGCATAAATCGAATGTCCGCTTAGAACTTGAGGGCCTTGGGCTTC |
| 10273-e2-F | TGACCACACGTCCTCCTGGTAG |
| 10273-e2-R | CTTTATGTACGCAGATGAATTCAAGACCTACCAGGAACAGCTTGGG |
| 10273-e1-F | CTTGAATTCATCTGCGTACATAAAG |
| 10273-e1-R | ATGGCTCAACTCGACACT |
| 10273-MCS1-F | GGTCGAGAGTGTCGAGTTGAGCCATCGATACTAGTGCGGCCGCCC |
| 6231-MCS2-R | ACTGGAAGAGCGTTAGAAAACCCATGTCGACGCCCGGGCCCTATA |
| 6231-F | ATGGGTTTTCTAACGCTCTTCCAGT |
| 6231-R | CGCCTCTATAAGGCAGCGATGC |
| 6231-MCS2-F | GGGGCATCGCTGCCTTATAGAGGCGGAACAGAAGTTGATTTCCGAAGAAG |
| 10273-MCS1-R | CAGCTACCAGGAGGACGTGTGGTCAGATTACAAGGATGACGACGATAAGA |
| pESC-HIS-F | CCTCTTCGCTATTACGCCAGCTGAA |
| pESC-HIS-R | TAGTCCTGTCGGGTTTCGCCAC |
| Leu101-F | TTCTACCATGCGGCGGCGCCGCCCAA |
| Leu101-R | TTGGGCGGCGCCGCCGCATGGTAGAA |
| Ser204-F | AGCAGATAGCGTCGTTGGCACC |
| Ser204-R | GGTGCCAACGACGCTATCTGCT |
| Arg210-F | TTGGCACCGGTGGCGACGACGTTCCCGTC |
| Arg210-R | GACGGGAACGTCGTCGCCACCGGTGCCAA |
| Arg287-F | AACGAAGCGGCGGCGTAC |
| Arg287-R | GTACGCCGCCGCTTCGTT |
| Thr296-F | GGAGCGACGCGACCGCAATCA |
| Thr296-R | TGATTGCGGTCGCGTCGCTCC |
| Glu346-F | GGTGATTAACGCGGCTTTGCGAT |
| Glu346-R | ATCGCAAAGCCGCGTTAATCACC |
| Arg349-F | CGAGGCTTTGGCGTTGTATACGG |
| Arg349-R | CCGTATACAACGCCAAAGCCTCG |
| Arg404-F | TACCCGGAGGCGTGGGAAAC |
| Arg404-R | GTTTCCCACGCCTCCGGGTA |
| Cys426-F | GGCACGCGCTGCGATTGG |
| Cys426-R | CCAATCGCAGCGCGTGCC |
| Phe468-F | GGAGATGAAGAGCGCGTTTCTTATG |
| Phe468-R | CATAAGAAACGCGCTCTTCATCTCC |
| Leu470-F | GAGCTTCTTTGCGATGGCGCCC |
| Leu470-R | GGGCGCCATCGCAAAGAAGCTC |
| pESC63-F | AGAAGCCACCTCGCCCAATGG |
| pESC63-R | CCATTGGGCGAGGTGGCTTCT |
| pESC59-F | CCAGAGCGGTGGTAGATCTTTC |
| pESC59-R | GAAAGATCTACCACCGCTCTGG |
| pESC72-F | GAAGCCACCTCGCCCAATGGTACCAACGATGTTCCCT |
| pESC72-R | AGGGAACATCGTTGGTACCATTGGGCGAGGTGGCTTC |
| 101-468-F | TATGGAGATGAAGAGCGCGTTTCTTATGGCGC |
| 101-468-R | GCGCCATAAGAAACGCGCTCTTCATCTCCATA |
| 101-470-F | ATGAAGAGCTTCTTTGCGATGGCGCCCAAG |
| 101-470-R | CTTGGGCGCCATCGCAAAGAAGCTCTTCAT |
| 468-470-F | ATGAAGAGCGCGTTTGCGATGGCGCCCAAG |
| 468-470-R | CTTGGGCGCCATCGCAAACGCGCTCTTCAT |

## Table S4. The FPKM of cytochrome P450s in HXN301 and PgedA-PtaI

| **Accession** | **HXN301** | **PgedA-PtaI** | | | |
| --- | --- | --- | --- | --- | --- |
|  | 1 day | 1 day | 2 day | 4 day | 6 day |
| **CYP-H6231** | 28.22179233 | 15.852687 | 1086.29126 | 3239.605306 | 3347.666015 |
| **CYP-H2247** | 1026.315023 | 338.48998 | 521.1945597 | 184.7313333 | 71.80744667 |
| **CYP-H3969** | 7.206887333 | 46.57601433 | 511.674184 | 418.3703917 | 325.9553223 |
| **CYP-H10138** | 168.3040213 | 174.8685913 | 315.8371173 | 155.767049 | 117.112854 |
| **CYP-H7972** | 177.4531507 | 237.21579 | 206.347285 | 162.6558127 | 97.56678233 |
| **CYP-H477** | 30.735124 | 165.8303553 | 142.5397743 | 56.355707 | 26.94962867 |
| **CYP-H3291** | 147.221334 | 54.44444633 | 123.7765603 | 42.67719267 | 36.62741733 |
| **CYP-H8017** | 94.410065 | 119.5693487 | 114.21286 | 117.3375547 | 85.13493333 |
| **CYP-H5224** | 126.8437147 | 112.126086 | 110.1516063 | 120.7100933 | 135.9128877 |
| **CYP-H3982** | 0.181309 | 0.393619667 | 78.27900433 | 171.1738787 | 193.2675933 |
| **CYP-H7481** | 384.7003987 | 89.68076333 | 77.501541 | 37.649346 | 24.94947167 |
| **CYP-H2234** | 57.76857767 | 63.02139533 | 70.86084267 | 83.04465233 | 57.67049767 |
| **CYP-H10634-s1** | 127.588679 | 48.98868833 | 46.24331667 | 31.108334 | 33.63846633 |
| **CYP-H2623** | 83.093778 | 30.926481 | 43.10874167 | 18.86845467 | 21.34148933 |
| **CYP-H6511** | 18.70806333 | 25.650709 | 27.02688533 | 16.88585333 | 12.87841967 |
| **CYP-H842** | 30.118681 | 27.75740033 | 26.201173 | 21.88049967 | 18.464156 |
| **CYP-H38** | 0.104418667 | 0.259754 | 25.34387067 | 36.90430067 | 18.53984633 |
| **CYP-H5570** | 11.02027233 | 4.978558 | 15.040289 | 25.062328 | 37.302793 |
| **CYP-H7042** | 35.07437233 | 11.384473 | 14.68895433 | 17.98260167 | 17.41263433 |
| **CYP-H4663** | 19.177426 | 10.88366 | 13.09327933 | 24.82044533 | 26.30022167 |
| **CYP-H328** | 4.632761667 | 18.25995533 | 11.606615 | 6.870208 | 10.744862 |
| **CYP-H2820** | 20.99126267 | 7.977077 | 10.63050567 | 7.948018 | 7.063632333 |
| **CYP-H4989** | 8.286978333 | 5.442532 | 10.11134567 | 25.048839 | 22.22924867 |

## Table S5. The CPRome of *Aspergillus terreus*

| **CPR** | **Annotation** |
| --- | --- |
| H10273 |  |
| H764 |  |
| H00121015 | Fused self-sufficient CPR |
| H001208920 | Fused self-sufficient CPR |
| H001217963 | Electron transfer flavoprotein β-subunit |
| H001218611 | Cytochrome b5 |
| H001215899 | Cytochrome b5 |

# Supporting Figures

## Fig. S1 LC-HRMS characterization of ω-hydroxyemodin, fallacinol, emodin, and physcion isolated from Pgpd-PtaI fermentation culture

## Fig. S2 HPLC analysis of emodin feeding assay in HXN301

## Fig. S3 PCR verification of the ΔgedA-ΔH6231, ΔgedA-ΔH2247, ΔgedA-ΔH3969 and PgedA-PtaI-ΔH6231

## Fig. S4 Production titers (g/L) of anthraquinone compounds by different genetic variants

(A) Comparison of ω-hydroxyemodin, emodin, and anthraquinone production by ΔgedA variants. (B) Comparison of emodin, physcion, and anthraquinone production by PgedA-Ptal variants. Compound titers (g/L) were determined by HPLC. Bars represent the mean values of three independent experiments, and error bars indicate standard deviation.

## Fig. S5 SDS-PAGE analysis of purification of AwOMT

## Fig. S6 PCR verification of the 3-EOMT, SamS, acetyl-CoA carboxylase (ACC) and EwMFS overexpression variants

## Fig. S7 Biosynthetic pathway of SAM in *A. terreus* and transcription levels of relevant genes

The metabolic pathway shows key intermediates and enzymes involved in SAM synthesis. Colored ovals indicate gene transcription levels: red (high transcription), blue (moderate transcription), and green (low or undetectable transcription). SAM (blue box) serves as the essential methyl donor for the *O*-methylation reaction.

## Fig. S8 Emodin, physcion and anthraquinone production by different genetic variants on day 7

Compound titers (mg compound/g pellet mycelia) were determined by HPLC. Bars represent the mean values of three independent experiments, and error bars indicate standard deviation.

## Fig. S9 Time course analysis of emodin (A) and physcion (B) production after feeding different SAM concentrations

Error bars represent standard deviations from three biological replicates.

## Fig. S10 Transmembrane domain prediction analysis of the EwMFS transporter protein using TMHMM 2.0

A

B

## Fig. S11 Analysis of metabolite production and compound distribution in strains expressing the EwMFS transporter

(**A**) Titers (mg compound/g pellet mycelia) of ω-hydroxyemodin, emodin, and total anthraquinones in ΔgedA and derivative strains on days 4 and 7. (**B**) Intracellular versus extracellular distribution (%) of emodin and physcion in the different genetic variants indicated. Error bars represent standard deviations from three independent experiments.

## Fig. S12 PCR verification of the protein fusion and co-expression variants

## Fig. S13 Analysis of emodin, physcion, and anthraquinone production levels and emodin-to-physcion transformation efficiency in different variants

The bar graph displays the titers of emodin (black), physcion (red), and anthraquinone (blue) in mg/g, while the line graph shows the emodin-to-physcion transformation rate (%) measured on day 4. Error bars indicate standard deviations from three independent experiments.

## Fig. S14 Cryo-scanning electron microscopy images showing spore morphology changes in P6231-PtaI, Pgpd-PtaI, and P6231-AwOMT strains

## Fig. S15 Overall structure of CYP-H6231 enzyme with docked emodin in the active site

Green stick represents emodin while yellow stick shows the heme porphyrin ring. The protein is displayed as a wheat ribbon.

## Fig. S16 PCR verification of CYP-H6231 mutants expressed in *Saccharomyces cerevisiae*

(**A**) Verification of heterologous expression of CYP-H6231 and CPR-H10273.

(**B**) Site-directed mutagenesis of CYP-H6231. The upper panel shows single-point mutants; the lower panel shows multiple-point mutants.

## Fig. S17 HPLC analysis of ω-hydroxyemodin production in *S. cerevisiae* strains expressing site-directed mutants of CYP-H6231

HPLC profiles of single-point (A and B) and combined-point (C) mutants of CYP-H6231. Wild-type CYP-H6231 (10273-6231), emodin, ω-hydroxyemodin, and vector-only control were included as references. The detection wavelength was 440 nm.

## Fig. S18 Multiple protein sequence alignment between CYP-H6231 and its analogous enzymes

Four conserved regions characteristic of CYP enzymes are highlighted in colored rectangles (blue, green and red), with key catalytic residues targeted for mutation indicated by purple arrows. Potential emodin-binding sites are marked with triangles and circles. Sequence analysis was conducted using Expresso through the T-COFFEE online service [5], and the figure was prepared by ESPript 3.0 [6].

## Fig. S19 LC-HRMS analysis of chrysophanol feeding assays in the HXN301 culture

## Fig. S20 Hypothetical biosynthesis pathway of the hydroxylation product fallacinol

(A) The proposal of hydroxylated anthraquinone derivatives biosynthetic pathway.

(B) Clarification of fallacinol biosynthesis by *in vitro* assay.

# Supporting References

1. Danielsen K, Aksnes DW: NMR-Study Of Some Anthraquinones From Rhubarb. Magn Reson Chem. 1992; 30**:**359-360 doi:DOI 10.1002/mrc.1260300414.

2. Cohen PA, Towers GHN: Anthraquinones And Phenanthroperylenequinones From Nephroma-Laevigatum. J Nat Products. 1995; 58**:**520-526 doi:DOI 10.1021/np50118a006.

3. Wang WL, Zhu TJ, Tao HW, Lu ZY, Fang YC, Gu QQ, Zhu WM: Two new cytotoxic quinone type compounds from the halotolerant fungus. J Antibiot. 2007; 60**:**603-607 doi:DOI 10.1038/ja.2007.77.

4. Qi F, Zhang W, Xue Y, Geng C, Jin Z, Li J, Guo Q, Huang X, Lu X: Microbial production of the plant-derived fungicide physcion. Metab Eng 2022; 74**:**130-138 doi:10.1016/j.ymben.2022.10.007.

5. Di Tommaso P, Moretti S, Xenarios I, Orobitg M, Montanyola A, Chang JM, Taly JF, Notredame C: T-Coffee: a web server for the multiple sequence alignment of protein and RNA sequences using structural information and homology extension. Nucleic Acids Res. 2011; 39**:**W13-W17 doi:10.1093/nar/gkr245.

6. Robert X, Gouet P: Deciphering key features in protein structures with the new ENDscript server. Nucleic Acids Res. 2014; 42**:**W320-W324 doi:10.1093/nar/gku316.
